# Cryo‐EM reconstruction of Type VI secretion system baseplate and sheath distal end

**DOI:** 10.15252/embj.201797103

**Published:** 2017-12-18

**Authors:** Sergey Nazarov, Johannes P Schneider, Maximilian Brackmann, Kenneth N Goldie, Henning Stahlberg, Marek Basler

**Affiliations:** ^1^ Focal Area Infection Biology Biozentrum, University of Basel Basel Switzerland; ^2^ Center for Cellular Imaging and NanoAnalytics Biozentrum, University of Basel Basel Switzerland; ^3^ Focal Area Structural Biology Biozentrum, University of Basel Basel Switzerland

**Keywords:** baseplate, contractile tails, cryo‐electron microscopy, sheath, Type VI secretion system, Microbiology, Virology & Host Pathogen Interaction, Structural Biology

## Abstract

The bacterial Type VI secretion system (T6SS) assembles from three major parts: a membrane complex that spans inner and outer membranes, a baseplate, and a sheath–tube polymer. The baseplate assembles around a tip complex with associated effectors and connects to the membrane complex by TssK. The baseplate assembly initiates sheath–tube polymerization, which in some organisms requires TssA. Here, we analyzed both ends of isolated non‐contractile *Vibrio cholerae* sheaths by cryo‐electron microscopy. Our analysis suggests that the baseplate, solved to an average 8.0 Å resolution, is composed of six subunits of TssE/F_2_/G and the baseplate periphery is decorated by six TssK trimers. The VgrG/PAAR tip complex in the center of the baseplate is surrounded by a cavity, which may accommodate up to ~450 kDa of effector proteins. The distal end of the sheath, resolved to an average 7.5 Å resolution, shows sixfold symmetry; however, its protein composition is unclear. Our structures provide an important step toward an atomic model of the complete T6SS assembly.

## Introduction

Bacteria use phage‐related contractile nanomachines to kill competition or manipulate the surrounding environment (Nakayama *et al*, 2000; Heymann *et al*, 2013; Shikuma *et al*, 2014; Ge *et al*, 2015). Evolutionarily related Type VI secretion system (T6SS) is used by bacteria to deliver proteins into both bacterial and eukaryotic cells (Mougous *et al*, 2006; Pukatzki *et al*, 2006; Hood *et al*, 2010; MacIntyre *et al*, 2010; Durand *et al*, 2014; Ho *et al*, 2014; Alcoforado Diniz *et al*, 2015; Hachani *et al*, 2016). The current model of T6SS biogenesis and mode of action is largely based on well‐understood phage assembly (Leiman *et al*, 2009; Lossi *et al*, 2011, 2013; Ho *et al*, 2014; Zoued *et al*, 2014; Clemens *et al*, 2015; Kudryashev *et al*, 2015; Cianfanelli *et al*, 2016). However, many important features are unknown mostly due to the lack of high‐resolution structural information of T6SS.

The whole T6SS was first observed at low resolution using cryo‐electron tomography (cryo‐ET) in *Vibrio cholerae* (Basler *et al*, 2012) and more recently in *Myxococcus xanthus* (Chang *et al*, 2017) and *Amoebophilus asiaticus* (Böck *et al*, 2017). The system is composed of a long sheath surrounding an inner tube attached to a baseplate and anchored to a membrane complex that spans both membranes (Brunet *et al*, 2014, 2015; Durand *et al*, 2015). T6SS biogenesis starts by formation of the membrane complex composed of TssJLM, which recruits TssK to connect a baseplate composed of TssEFG, as well as VgrG/PAAR spike and effectors (Brunet *et al*, 2015; Durand *et al*, 2015). The baseplate then serves as a nucleation platform for polymerization of the VipA/VipB (TssB/TssC) sheath and the inner Hcp tube (Zoued *et al*, 2016). Additionally, while one class of TssA proteins is required for baseplate assembly, another class of TssA proteins was shown to first bind to the baseplate and then stay associated with the distal end of the sheath during assembly (Planamente *et al*, 2016; Zoued *et al*, 2016).

The *V. cholerae* sheath subunits are interconnected by a mesh of VipA N‐terminal and VipB C‐terminal linkers (Kudryashev *et al*, 2015) similarly to T6SS sheath of *Francisella novicida* and R‐type pyocin sheath (Clemens *et al*, 2015; Ge *et al*, 2015). The sheath was shown to assemble across the entire width of a cell, and this allowed to use live‐cell fluorescence microscopy to study its dynamics and subcellular localization (Basler *et al*, 2012; Brunet *et al*, 2013; Gerc *et al*, 2015; Zoued *et al*, 2016; Vettiger *et al*, 2017). In *V. cholerae*, sheath assembly takes approximately 20 s (speed of assembly 30–40 nm/s) and is followed by fast contraction in less than 2 ms (speed of contraction higher than 800 μm/s) (Basler *et al*, 2012; Vettiger *et al*, 2017). Rapid sheath contraction propels the tip complex at the end of the inner tube formed from stacks of Hcp rings into the target cell periplasm or cytosol (Vettiger & Basler, 2016). In contrast to phages and many other contractile nanomachines, which translocate proteins only once by a single sheath contraction (Nakayama *et al*, 2000; Ge *et al*, 2015; Hu *et al*, 2015), the contracted T6SS sheath is disassembled by a cytosolic unfoldase ClpV or ClpB to allow for repeated protein secretion (Bönemann *et al*, 2009; Pietrosiuk *et al*, 2011; Basler & Mekalanos, 2012; Basler *et al*, 2012; Kapitein *et al*, 2013; Förster *et al*, 2014; Brodmann *et al*, 2017).

The membrane complex seems to be stable at least in some organisms (Durand *et al*, 2015; Santin & Cascales, 2017); however, the whole T6SS was so far impossible to isolate from cells for *in vitro* studies. This is likely due to the highly dynamic behavior of T6SS, and it suggests that cell disruption triggers the sheath contraction, which in turn may lead to baseplate disassembly. Here, we analyzed both ends of a non‐contractile T6SS sheath mutant, which has three amino acids inserted into the N‐terminal VipA linker (VipA‐N3) and was shown recently to co‐purify with many components of T6SS baseplate (Brackmann *et al*, 2017; Wang *et al*, 2017). On one end, the sheath is attached to a 2.2‐MDa complex with flattened cone geometry and dimensions of 240 × 124 Å. This complex resembles the phage T4 inner baseplate with a central sharp spike and is decorated by six protruding trimeric proteins. An extended‐like sheath with a tube is docked onto the baseplate, and the overall structure resembles a typical contractile tail of bacteriophages. The opposite end of the sheath is covered with a flattened starlike complex with dimensions 190 × 67 Å and an estimated mass of 540 kDa and could represent either VipA/VipB sheath subunits without Hcp ring in an unusual conformation, or a cap composed of twelve copies of TssA protein. Together, our results indicate that blocking sheath contraction allows isolation of a largely intact T6SS and this may provide a path toward a detailed atomic model of the whole T6SS in a “ready‐to‐fire” conformation.

## Results

### Analysis of protein complexes associated with the ends of non‐contractile sheaths

**Figure 1:**
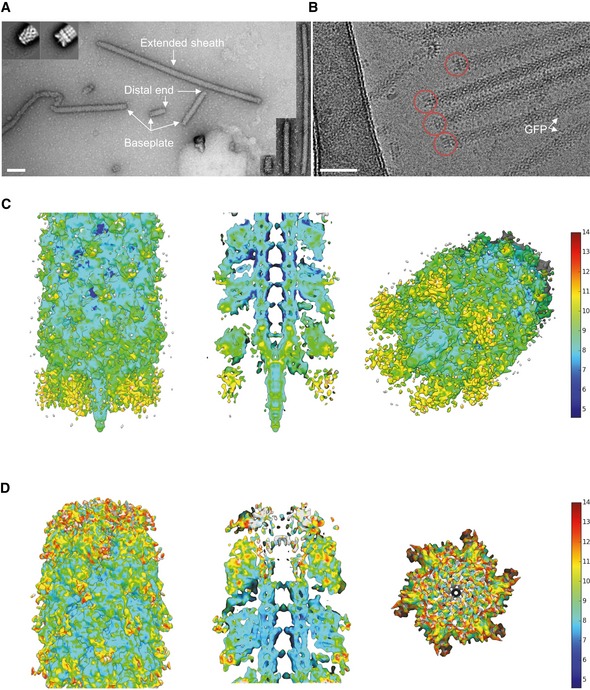
Raw images and cryo‐EM structures of T6SS baseplate and distal‐end complexes A raw negative‐stained image of VipA‐N3 mutant sheath (scale bar: 100 nm). Extended sheath, baseplate, and distal end are highlighted with white arrows. Right insets: examples of T6SS assemblies of different lengths (shortest ˜5 sheath rings, longest ˜220 rings). Upper left insets: representative reference‐free 2D class averages of baseplate (right) and distal‐end (left) particles, extracted from negative‐stained images.A raw cryo‐EM image of VipA‐N3 mutant (scale bar: 50 nm). Baseplate reconstruction was calculated for area highlighted with red circles.Side, cutaway, and tilted views of the sheath–baseplate cryo‐EM reconstruction. 3D visualizations were rendered using UCSF Chimera. Baseplate model colored according to the local resolution variation, shown in the color bar on the right in Angstroms. Maps are shown at the contour level of 1.5σ.Side, cutaway, and top views of the sheath–distal‐end cryo‐EM reconstruction. 3D visualizations were rendered using UCSF Chimera. Distal‐end model colored according to the local resolution variation, shown in the color bar on the right in Angstroms. Maps are shown at the contour level of 1.5σ.

Non‐contractile sheaths of *V. cholerae* were shown to be associated with certain baseplate components (Brackmann *et al*, 2017). We isolated the VipA‐N3 sheath variant (3‐amino acid insertion into the N‐terminal VipA linker, tagged with msfGFP) from cells and purified it by ultracentrifugation as described previously (Kudryashev *et al*, 2015). Negative stain electron microscopy showed sheath assemblies of various lengths in apparently extended conformations (Fig 1A). On average, 39% of these sheath assemblies were decorated with distinct protein complexes at one of the two ends. Further image analysis and averaging revealed that one end resembles a phage‐related baseplate and the second distal end has a distinct structure resembling a cap (Fig 1A).

**Figure EV1:**
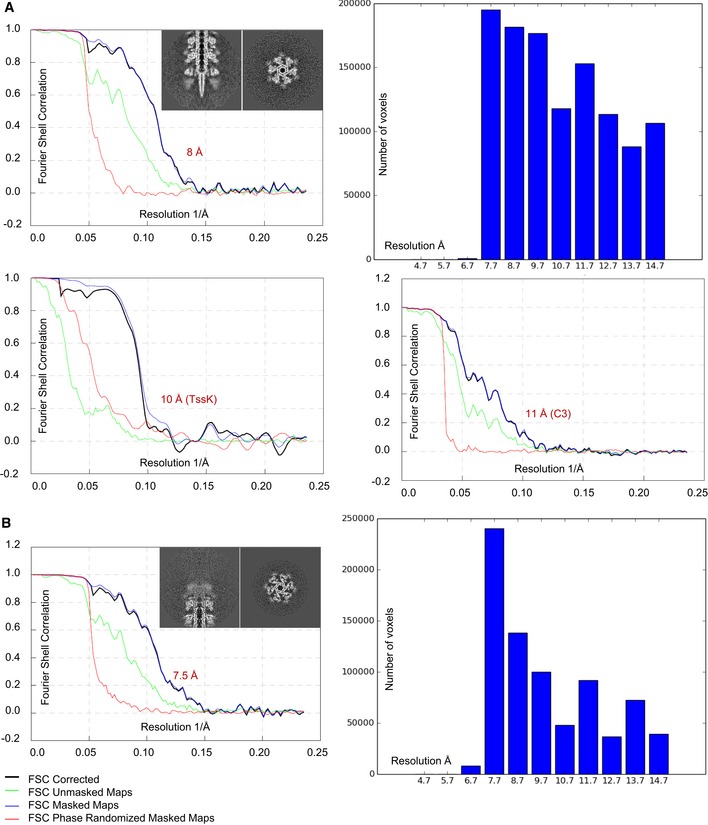
Analysis of raw data, estimation of resolution Representative slices through the raw sheath–baseplate reconstruction. Gold‐standard FSC curves calculated for the sixfold‐averaged (top left) and threefold‐averaged (bottom right) baseplate reconstructions and for the TssK reconstruction (bottom left). Histogram of the local resolution estimation of the baseplate reconstruction (top right).Representative slices through the raw sheath–distal‐end reconstruction. Left: Gold‐standard FSC curves calculated for the sixfold‐averaged distal‐end reconstruction. Right: Histogram of the local resolution estimation of the distal‐end reconstruction.

To increase the resolution, we analyzed the non‐contractile sheath sample by cryo‐electron microscopy using Titan Krios and a K2 Summit direct electron detector in counting mode, operated at 300 kV and at a nominal magnification of 130,000×, corresponding to a calibrated pixel size of 1.06 Å. Overall, we collected 6,603 movies, performed specimen motion correction using MotionCorr2 (Zheng *et al*, 2017), and selected 21,446 particles from both ends of the non‐contractile sheath assemblies. 8,309 baseplates and distal ends were identified after 2D classification, and 2,660 baseplates and 3,710 distal ends were further processed by RELION1.4 (Scheres, 2012) to obtain 3D density (Appendix Table S1). Based on available structural data of other contractile tail‐like structures (Schwarzer *et al*, 2012; Heymann *et al*, 2013; Shikuma *et al*, 2014; Ge *et al*, 2015; Nováček *et al*, 2016; Taylor *et al*, 2016), we applied a sixfold symmetry (C6) during refinement of the baseplate and the distal end. The final resolution was estimated to be on average 8.7 Å (local resolution distribution from 7.2 to 14.2 Å) for the baseplate and 7.5 Å (from 6.7 to 13.7 Å) for the distal end (Fig EV1A). For the baseplate reconstruction, we also tested threefold (C3) symmetry during the refinement step using symmetry relaxation from C6 to C3. This resulted in a 11 Å resolution reconstruction (Appendix Fig S1A) with the same overall baseplate morphology, indicating that the C6 symmetry choice is correct and helps to improve the final resolution of the baseplate reconstruction.

**Figure EV2:**
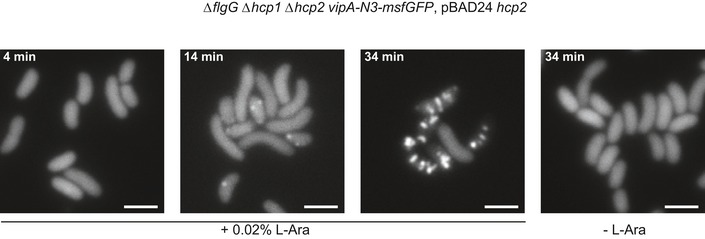
Sheath formation in Hcp‐limited cells Fluorescence timelapse images of *Vibrio cholerae vipA‐N3‐msfGFP* in *hcp1/2* mutant background, complemented with *hcp* expressed from L‐arabinose‐inducible vector pBAD24. Scale bars are 2 μm.

**Figure EV3:**
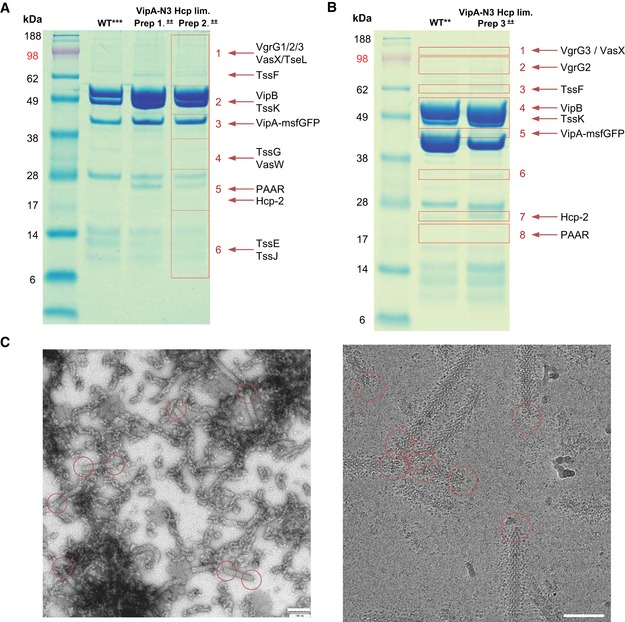
Mass spectrometry and EM analysis of preparations from Hcp‐limited cells SDS–PAGE of purified sheaths: wild‐type Hcp‐limited prep, and (Hcp‐limited + VipA‐N3) Prep1 and Prep2. Areas 1–6 cut from gel and sent for mass spectrometry. Detected structural proteins are shown on the right.SDS–PAGE of purified sheaths: wild‐type and (Hcp‐limited + VipA‐N3) Prep3. Areas 1–8 cut from gel and sent for mass spectrometry. Detected structural proteins are shown on the right.Raw negative‐stained and cryo‐EM images of sample from (A) (Prep2). Scale bars are 100 nm. Representative baseplates and distal ends in the field of view are outlined with red circles.

The micrographs of non‐contractile sheaths are mostly covered with long sheaths (Fig 1B), and this decreases the number of clearly observed sheath ends. It was recently shown that limiting Hcp availability in *V. cholerae* results in assembly of short, dynamic, and functional sheaths (Vettiger & Basler, 2016). In an attempt to increase the speed of data acquisition, we expressed limited amount of Hcp from an inducible pBAD24‐*hcp* plasmid in the VipA‐N3‐msfGFP‐expressing strain lacking both *hcp1* and *hcp2* genes (Fig EV2). We then purified the non‐contractile sheaths as previously (Fig EV3) and characterized the sample by mass spectrometry. This showed that the sheaths prepared from Hcp‐limited cells are associated with TssE,F,G,K, VgrG1/2/3, and PAAR (Fig EV3A and B, Tables EV1, EV2 and EV3) similarly to the previous preparations. We collected 2,599 cryo‐EM movies and selected 1,127 particles for the baseplate and 2,114 for the distal end. Combining this dataset with the dataset described above and selecting the best particles for the final 3D refinement, the final resolution of the baseplate increased from 8.7 to 8.0 Å (Fig EV1A), which improved overall interpretation of the protein density map. The resolution of the distal‐end reconstruction did not improve, indicating dynamic nature of the complex. However, we also detected aberrant sheath fragments (Fig EV3C), which complicated single‐particle analysis, and thus, these samples failed to provide a major increase in data acquisition rate.

### Central spike and cavity for effector proteins

**Figure 2:**
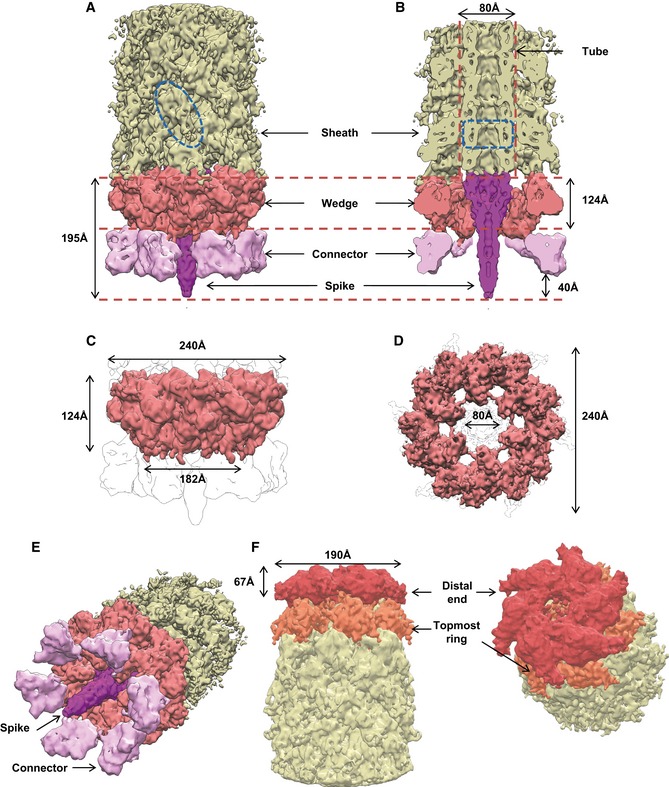
Overall dimensions, segmentation, and morphology of the baseplate and the distal end A, BSide and cutaway views of the sheath–baseplate cryo‐EM reconstruction. The partially disordered connector density was replaced by the locally refined reconstruction calculated separately. The map is segmented, and protein complexes are colored and highlighted with black arrows. One VipA/VipB sheath subunit and one Hcp ring are outlined in blue.C, DSide and bottom views of the putative baseplate density (1.15 MDa), highlighted in red in the sheath–baseplate cryo‐EM reconstruction in (A, B).ETilted view of (A) with connector and spike densities highlighted with black arrows.FCutaway and tilted views of the sheath–distal‐end cryo‐EM reconstruction, locally filtered to the estimated resolution. The map is segmented and colored according to putative protein complexes.

The cryo‐EM reconstruction of the baseplate region revealed a 238‐Å‐wide sheath–tube assembly in its extended‐like state attached to a compact hexagonal flattened conelike baseplate with a sharp tip in the center (Figs 1C and 2A–E). Overall dimensions of the baseplate are 182 Å in the narrow part, 240 Å in the broad part below the sheath–tube, and 195 Å in height including the protruding tip complex (Fig 2A and B). The central tip protrudes ~40 Å below base of the cone and clearly resembles a central spike present in all baseplates of phages with contractile tails (Browning *et al*, 2012; Leiman & Shneider, 2012; Schwarzer *et al*, 2012; Harada *et al*, 2013; Shneider *et al*, 2013; Habann *et al*, 2014; Taylor *et al*, 2016).

**Figure 3:**
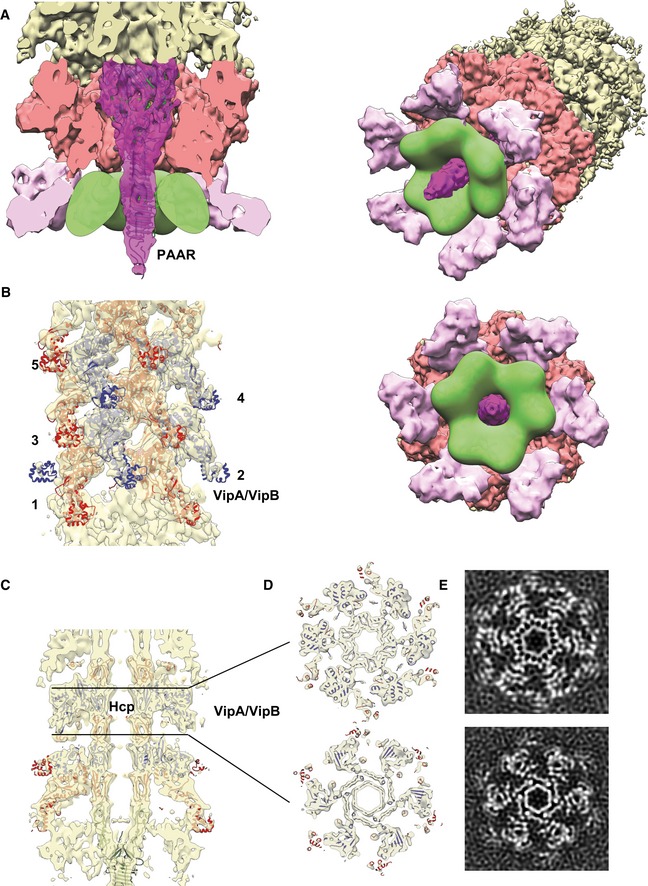
Central spike, tube, and sheath densities with a cavity for effector proteins Cutaway view of the density corresponding to the baseplate and spike with fitted X‐ray crystallographic structures of VgrG‐1 trimer (PDB 4MTK) and PAAR monomer (PDB 4JIV). Putative cavity for T6SS effectors is outlined in green.Side view of the density corresponding to the sheath next to the baseplate fitted with the atomic model of the VipA‐N3 sheath–tube (PDB 5MXN). In panels (B–D), odd sheath rings are colored in red, even in blue. Sheath rings are numbered starting from the first ring next to the baseplate.Side cutaway view of the baseplate reconstruction with the fitted atomic model of VipA‐N3 sheath–tube (PDB 5MXN).Top cutaway views of (C) with the fitted atomic model of VipA‐N3 sheath–tube (PDB 5MXN).Slices through the raw sheath–baseplate reconstruction, as in (D).

The T6SS spike complex is proposed to be formed by a VgrG trimer and a single PAAR protein (Leiman *et al*, 2009; Shneider *et al*, 2013). The central spike density has indeed well‐resolved features in the threefold symmetry reconstruction (Appendix Fig S1A). The available X‐ray crystallographic structure of PA0091 VgrG‐1 trimer (PDB 4MTK) and PAAR monomer (PDB 4JIV) fits well into both C3 (correlation coefficient (CC) = 0.93) and C6 reconstructions (CC = 0.88) (Fig 3A and Appendix Table S2). This fit was used to calculate the protein density volume‐to‐mass coefficient to estimate the mass of other protein complexes described below.

T6SS effectors can be attached to the spike by several different mechanisms (Shneider *et al*, 2013). Mass spectrometry analysis identified T6SS effectors VasX and TseL associated with the non‐contractile sheaths (Tables EV1, EV2 and EV3). However, the cryo‐EM reconstruction of VgrG/PAAR spike lacks any additional densities of possible effectors, and no density is observed inside VgrG or Hcp tube (Fig 3A and C–E). It is likely that each spike complex is decorated with a different set of effectors, and thus, the structures of the individual spikes are heterogeneous and the effector protein densities are missing after single‐particle averaging. We estimate that the volume of the internal cavity formed around the central spike by the baseplate periphery is ~1,100 × 10^3^ Å^3^, which would be enough to accommodate multiple effectors with the overall molecular weight up to ~450 kDa, or three copies of ~150‐kDa effector (Fig 3A).

### Baseplate wedge and connection to the membrane complex

The central spike is surrounded by six copies of a structure resembling a phage baseplate wedge (Leiman & Shneider, 2012; Büttner *et al*, 2016; Taylor *et al*, 2016; Fig 2) and is decorated by a protein density, which is likely a connector between the baseplate and the membrane complex (Fig 2A–E). Overall, the T6SS baseplate resembles so‐called minimal baseplate of contractile tail‐like structures and is similar to the T4 phage inner baseplate in its pre‐injection state (Appendix Fig S2; Leiman & Shneider, 2012; Taylor *et al*, 2016).

We estimate the molecular mass of a single baseplate wedge to be 191.2 kDa and 1.15 MDa for the overall dome‐shaped assembly (Fig 2 and Table 1). Three conserved baseplate components, TssE, TssF, and TssG, share structural homology with T4 inner baseplate proteins: sheath initiator gp25, sheath platform gp6, and linker gp7 (Leiman *et al*, 2010; Taylor *et al*, 2016). Together, these proteins have been proposed to form a wedge subunit intermediate with a possible stoichiometry TssE–(TssF)_2_–TssG (Taylor *et al*, 2016). Molecular mass of the TssE–(TssF)_2_–TssG complex of *V. cholerae* based on their primary sequence is 189.2 kDa, which is in a good agreement with the mass estimation based on the baseplate reconstruction (Table 1).

**Table 1:** Assignment of T6SS baseplate and distal‐end proteins based on volume‐to‐mass coefficient calculated for fitted X‐ray crystallographic structures of VgrG‐1/PAAR (PDB 4MTK/4JIV)

Protein	Copies per assembly	Mass_EM_, kDa	Mass_seq_, kDa		Function	Homologues (T4)
TssE	6	192 for a single wedge	16	189 for a single wedge	Wedge	gp25
TssF	12		67		Wedge	gp6
TssG	6		38		Wedge	gp7/gp53
TssK	18	58	50		Connector	gp10
TssA_1_ or VipA/VipB	12 or 6	90	53 or 74		Cap or Topmost sheath	–

**Figure 4:**
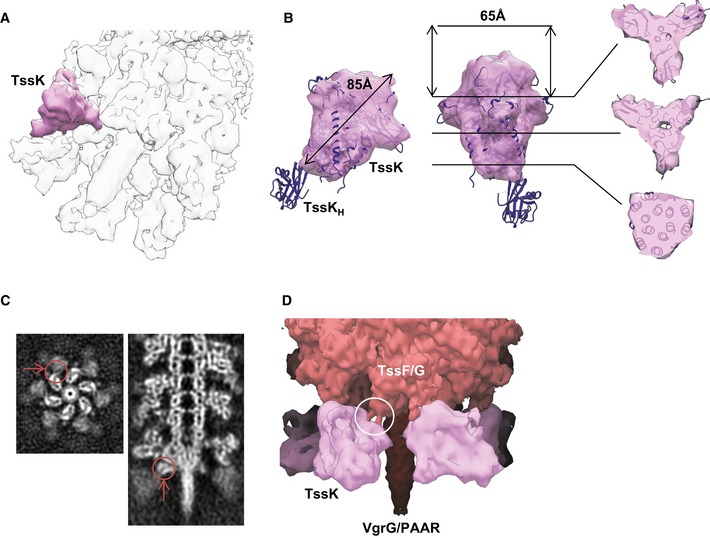
Trimeric connector protein and its interaction with the wedge Tilted view of the composite baseplate cryo‐EM reconstruction. One connector protein is superimposed with the locally refined reconstruction shown in pink.Orthogonal side views and slices perpendicular to the threefold axis through the locally refined reconstruction of the trimeric connector protein density. X‐ray crystallographic structure of EAEC TssK protein (PDB 5M30) was fitted into connector reconstruction except for the disordered C‐terminal head domain.Top and side slices through the raw sheath–baseplate reconstruction. Putative interface between connector and wedge highlighted with red arrows and circles.Side view of the sheath–baseplate cryo‐EM reconstruction. The partially disordered connector density was replaced by the locally refined reconstruction. Putative coiled‐coil interface between connector and wedge highlighted with white circle. Putative localization of the baseplate proteins is indicated.

**Figure EV4:**
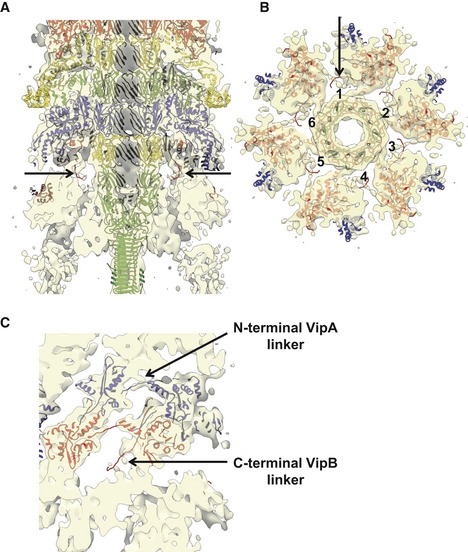
Baseplate–sheath connection Side cutaway view of the baseplate reconstruction with fitted atomic model of VipA‐N3 sheath–tube (PDB 5MXN) and VgrG‐1 trimer (PDB 4MTK) and PAAR monomer (PDB 4JIV). Putative C‐terminal VipB linkers are highlighted with black arrows.Bottom cutaway view of (A) below the first sheath ring. Six putative C‐terminal VipB linkers are numbered, and one is highlighted with black arrow.Enlarged side cutaway view of (A) of the first and second sheath rings with fitted atomic model of VipA‐N3 sheath–tube (PDB 5MXN). Putative C‐terminal VipB and N‐terminal VipA linkers are highlighted with arrows. N‐terminal linker connects to the neighboring protomer in the same ring.

Each baseplate wedge subunit is decorated with a ~85 Å long and ~65 Å wide protein density forming a continuation of the baseplate wedge flattened cone (Figs 2A, B, E and 4A, B, D). This protein density was initially resolved to the local resolution of only 14 Å, indicating that this protein complex or its domains are flexible and may be partially disordered. Another possibility is that this protein complex is occasionally present in less than six copies per baseplate, potentially due to instability during purification and the applied sixfold (C6) symmetry thus decreases the final resolution. To account for possible protein flexibility and low occupancy, we relaxed symmetry of the refined baseplate particles from C6 to C1 and performed 3D classification without alignment within a small mask around this decoration protein. The best 3D class was subsequently used for further focused 3D refinement, which increased the resolution to 10 Å and revealed clear trimeric features (Figs EV1 and 4A and B). Therefore, we estimate that this complex is composed of trimer of a 58‐kDa protein (Table 1).

TssK was shown to form trimers in solution, it binds membrane complex proteins TssL/TssM as well as baseplate proteins TssF/TssG, and its spot localization correlates with T6SS activity (English *et al*, 2014; Brunet *et al*, 2015; Durand *et al*, 2015; Nguyen *et al*, 2017). Additionally, no wedge intermediate TssE–(TssF)_2_–TssG was formed in the absence of TssK (Taylor *et al*, 2016). Taking these previous observations into account, the trimeric protein density on the periphery of the baseplate wedge is therefore likely formed by the 50‐kDa TssK protein (Table 1; Figs 2A, B, E and 4A and B). Indeed, EAEC TssK protein structure (PDB 5M30) fits with a good agreement into our reconstruction (CC = 0.92) except for disordered C‐terminal head domain (Fig 4B).

Each copy of the TssK protein complex has a threefold rotational axis oriented with ~48° relative to the baseplate sixfold axis with a left‐handed twist and is thus similar to the domain IV of T4 peripheral baseplate protein gp10 (Appendix Fig S1). We suggest that TssK is likely interacting with TssG (MW = 38.4 kDa), which shares limited homology with T4 gp7 protein forming a link between the inner baseplate and the peripheral tail fiber network (Leiman *et al*, 2010; Taylor *et al*, 2016). TssK is also connected to the inner part of the baseplate wedge, likely the TssF dimer, through two fingerlike links (Fig 4C and D). Each link is about 22 Å long and 11 Å in diameter, suggesting that it may be formed by an α‐helix or a β‐hairpin loop. Together, those two connections would fix the TssK trimer to the baseplate wedge.

### Sheath–tube attachment to the baseplate

The spike complex is predicted to be an extension of the Hcp tube, which is surrounded by the VipA/VipB sheath (Wang *et al*, 2017). Indeed, the central density resembles the structure of an Hcp tube and seems to be uniform from the first ring docked on top of the VgrG/PAAR spike (Fig 3C), suggesting that no additional proteins are in between the VgrG trimer and the Hcp tube. This is in an agreement with the fact that T6SS gene clusters lack orthologues of tubelike proteins such as gp48 and gp54 found between the spike and the tube in T4 phage (Taylor *et al*, 2016).

The structure of the extended sheath (PDB 5MNX) fits into the density just above the baseplate with CC = 0.87 (Fig 3B–E and Appendix Table S2), suggesting that this density represents the first ring of the sheath. An almost identical density, representing the second sheath ring, is 36.4 Å above and is twisted by 23.9 degrees, which is similar to the helical parameters determined for the extended sheath (37.8 Å rise and 23.5 degrees twist) (Wang *et al*, 2017). The density of the second ring correlates with the sheath‐tube ring structure to CC = 0.86, further rings then to CC = 0.89 (Appendix Table S2).

**Figure 5:**
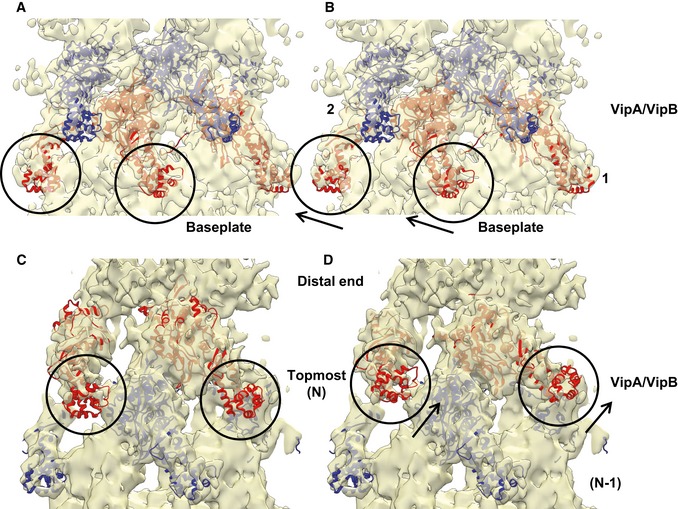
Rearrangements of the sheath rings next to the baseplate and the distal end Side view of the density corresponding to the sheath next to the baseplate fitted with the atomic model of the VipA‐N3 sheath–tube (PDB 5MXN).Same as (A), but with refitted sheath domain of the VipA‐N3 sheath–tube (PDB 5MXN). Differences between original and refitted sheath domains are outlined with black circles and arrows. Ring numbers 1 and 2 correspond to the first and second rings above the baseplate wedge.Side view of the density corresponding to the sheath next to the distal end fitted with the atomic model of the VipA‐N3 sheath–tube (PDB 5MXN).Same as (C), but with refitted sheath domain of the VipA‐N3 sheath–tube (PDB 5MXN). Differences between original and refitted sheath domains are outlined with black circles and arrows. Ring numbers N and (N‐1) correspond to the topmost ring and the previous ring.

Differences in correlation coefficients may be explained by slight changes in the conformation that the outer Domain 3 of the first and second sheath rings adopts due to interactions with the baseplate (Fig 5A and B). Those interactions could be important for preventing ClpV‐mediated sheath unfolding before contraction (Wang *et al*, 2017). One interface between the wedge and the adjacent sheath is between Domain 3 and the upper peripheral part of the wedge formed by TssF and TssG (Figs 3C and EV4A and C). This would be similar to T4 phage, where the external domain of the first sheath subunit gp18 potentially interacts with gp6/gp53 wedge proteins (Aksyuk *et al*, 2009; Taylor *et al*, 2016).

In the sheath, each subunit is connected by two β‐stranded linkers to two adjacent subunits in the ring closer to the baseplate (Clemens *et al*, 2015; Ge *et al*, 2015; Kudryashev *et al*, 2015). Because of the structural homology between TssE and sheath inner Domain 1, TssE was proposed to accept the linkers from the first sheath ring and thus connect sheath to the baseplate (Kudryashev *et al*, 2015) similarly to TssE homolog gp25 in T4 phage (Taylor *et al*, 2016). However, the subunits of the non‐contractile VipA‐N3 mutant sheath are connected to the previous sheath ring only through VipB linker and the elongated VipA linker connects to a neighboring subunit on the same sheath ring (Wang *et al*, 2017). This also means that the first sheath ring can only be connected to the baseplate by six linkers and not by twelve as in the wild‐type assembly or in the T4 phage (Kudryashev *et al*, 2015; Taylor *et al*, 2016). Even though our reconstruction lacks the resolution needed to resolve details of sheath–baseplate connection, there is a density originating from a sheath protomer likely corresponding to the VipB C‐terminal linker, which is most likely connected to TssE in the wedge (Fig EV4). However, we would expect to detect a density corresponding to TssE more clearly. It is possible that the absence of the second VipA linker destabilizes TssE and its density is thus less resolved.

### Overall structure of the distal end

**Figure EV5:**
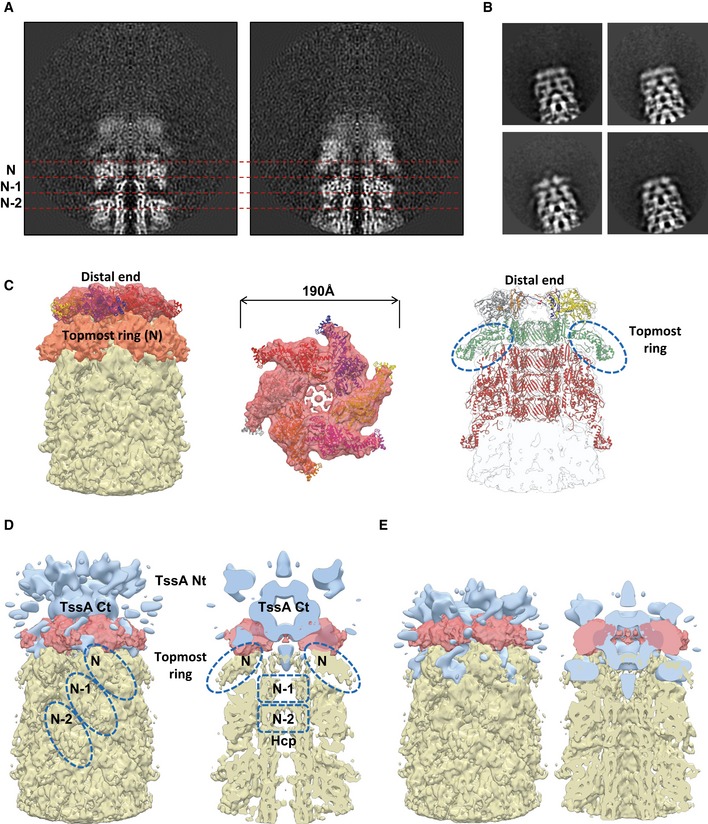
Morphology of the sheath distal end ATwo orthogonal slices through the raw sheath–distal‐end reconstruction shown. Ring numbers N, (N−1), and (N−2) correspond to the topmost ring and the two previous rings, and are separated by dashed red lines.BRepresentative reference‐free 2D class averages of the distal‐end particles, extracted from cryo‐EM images.CLeft and center: Side and top views of putative docking of refitted sheath domain of the VipA‐N3 sheath–tube (PDB 5MXN) into distal end. Right: Putative pseudo atomic model of the distal end with refitted sheath domain of the VipA‐N3 sheath–tube, refitted topmost sheath–tube ring and three rings below (PDB 5MXN). Topmost sheath rings are outlined with dashed blue circles.D, EPutative dockings of low‐resolution EM reconstruction EAEC TssA (EMD‐3282) into distal end of T6SS. Topmost sheath ring N, two previous sheath rings (N‐1) and (N‐2), and two tube rings (N‐1) and (N‐2) are outlined with dashed blue circles.

In our reconstruction, the side of the non‐contractile sheath that is opposite to the baseplate is clearly distinct from the adjacent VipA/VipB sheath and Hcp tube rings (Figs 1D, 2F–G, and EV5). The sheath and tube densities were resolved to 7.5 Å resolution; however, the distal‐end density was partially disordered. This density can be further split along the long sheath axis into two layers. The first layer was resolved to 14 Å resolution (shown in red in Figs 2E and EV5C), and the second layer was resolved to 10 Å resolution at the Hcp tube part and to 12 Å resolution at the VipA/VipB sheath periphery (orange in Figs 2E and EV5C), while rings below are resolved to 7.5 Å resolution as predicted by ResMap (Fig 1D; Kucukelbir *et al*, 2014). Six sheath subunits can be fitted into the layer that is next to the end of the regular sheath–tube polymer with a correlation coefficient 0.86, suggesting that this layer is composed of sheath–tube ring (topmost ring in Fig EV5C). However, the overall topology is different as the sheath subunits are rotated ~21 degree around the sheath axis and appear more flattened compared to the other sheath rings (Fig 5C and D). The layer at the very end of the complex is distinct and appears to be formed by six 90‐kDa large protein complexes (Table 1; Figs 2F and EV5).

In *Escherichia coli*, TssA forms dodecamers colocalizing with the end of an assembling sheath that is distal from the baseplate, and it was proposed to prime and coordinate sheath–tube polymerization (Zoued *et al*, 2016). This suggests that the complex detected on the very end of the sheath could be formed by a homolog of *E. coli* TssA. *V. cholerae* has two TssA proteins, TssA_1_ (locus name VCA0119) and TssA_2_ (locus name VCA0121). TssA_1_ is a close homolog of the *E. coli* TssA as it is also composed of an ImpA_N domain (PF06812) followed by T6SS_VasJ domain (PF16989) while TssA_2_ only has the ImpA_N domain. TssA_1_ has a molecular weight of 53.1 kDa and thus would likely form a dodecamer with a size close to 640 kDa. The fact that only approximately 540‐kDa complex is detected at the end of the isolated sheaths could be explained by low resolution and partial disordering of the protein complex. However, the complex at the sheath end aligns poorly with the structure of the *E. coli* TssA dodecamer (EMD‐3282) (Fig EV5D and E). This could be due to differences between TssA assemblies in solution and bound to sheath or could represent an actual difference in the overall structures of the *V. cholerae* TssA_1_ and *E. coli* TssA.

It is important to note that only small amounts of TssA_1_ protein were detected in our sheath preparations (Datasets EV4, EV5, EV6 and EV7), and therefore, it is also possible that the distal‐end density is composed of sheath subunits (74 kDa each) organized into flat starlike complex without the central Hcp hexamer. Indeed, sheath subunits fit the density with CC = 0.82 (Fig EV5C). The complex at the distal sheath end could therefore be a stable assembly intermediate rather than a cap composed of TssA_1_ protein.

## Discussion

Blocking sheath contraction helped to stabilize the T6SS and allowed us to isolate sheath with an attached baseplate and possibly a cap out of *V. cholerae* cells for high‐resolution cryo‐electron microscopy analysis. Although the 8 Å resolution of the baseplate reconstruction prevents precise segmentation of individual components, the overall baseplate structure clearly resembles T4 inner baseplate and represents the simplest type of baseplates conserved among other contractile tail‐like systems (Leiman & Shneider, 2012; Büttner *et al*, 2016; Taylor *et al*, 2016).

**Figure EV6:**
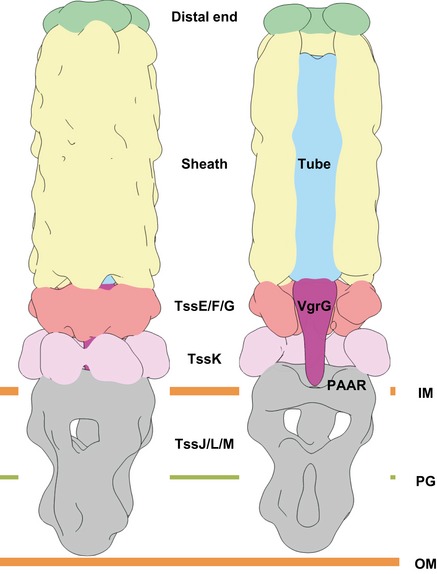
Schematic representation of the assembled T6SS Side and cutaway views of the map, composed of the overlapping sixfold‐averaged sheath baseplate, sixfold‐averaged sheath–distal end, and fivefold‐averaged membrane complex (EMD‐2927) reconstructions, strongly filtered with Gaussian filter to 10σ. The map is colored according to putative protein complexes.

The previous cryo‐ET studies of *V. cholerae*,* M. xanthus,* and *A. asiaticus* showed that T6SS sheath–tube complex is connected to the cell envelope through a cone‐shaped density (Basler *et al*, 2012; Böck *et al*, 2017; Chang *et al*, 2017). In our baseplate reconstruction, the periphery of the wedge likely composed of TssE/F/G is decorated by six copies of a trimeric protein TssK. It remains to be seen how six TssK trimers connect to the membrane complex with an apparent fivefold symmetry (Durand *et al*, 2015); however, it is possible that whole TssK or its parts are flexible and have certain degree of freedom to move and adopt to a structure with a different symmetry (Fig EV6). Recently solved structure of EAEC TssK protein (Nguyen *et al*, 2017) indeed revealed partially disordered C‐terminal head domain, which indicates its flexibility. C‐terminal head domain has been shown to interact with cytoplasmic domains of TssL and TssM proteins from membrane complex. The flexibility of TssK C‐terminus probably provides more degree of freedom for the baseplate–membrane complex formation.

The signal to trigger sheath contraction is currently unknown for T6SS; however, in phages, the conformational switch of the baseplate triggers contraction of the assembled sheath, which then pushes the rigid tube with the sharp tip through the target cell membranes attached by tail fibers (Browning *et al*, 2012; Leiman & Shneider, 2012; Hu *et al*, 2015; Taylor *et al*, 2016). The signal for the phage baseplate component rearrangement comes from the attachment of the tail fibers to a target cell (Hu *et al*, 2015). Similarly, cell–cell contact could be a trigger for T6SS sheath contraction. In that case, the membrane complex could induce changes in TssK structure, which would propagate through its interaction with TssE/F/G wedge to trigger sheath contraction. However, live‐cell imaging of T6SS sheath dynamics in *V. cholerae* provided no evidence for a triggering mechanism based on cell–cell contact (Basler & Mekalanos, 2012; Basler *et al*, 2012). Conversely, it is possible that rearrangement of baseplate proteins prior to sheath contraction changes TssK conformation, and this results in opening of the connected membrane complex to allow passage of the cargo‐loaded spike and tube. Such membrane complex rearrangement was indeed proposed based on the structure of the isolated TssJLM membrane complex (Durand *et al*, 2015).

The cavity around VgrG/PAAR spike is ~1,110 × 10^3^ Å^3^ large and may accommodate up to ~450‐kDa large proteins, or three copies of up to ~150‐kDa protein. This is consistent with sizes of known effectors: 129‐kDa VgrG‐1, 113‐kDa VgrG‐3, or 155‐kDa pair VasX‐VasW (Dong *et al*, 2013; Joshi *et al*, 2017). Since 77‐kDa VgrG‐2 with no extension domain is required for T6SS assembly (Appendix Fig S3; Pukatzki *et al*, 2007), there is also enough space for effectors interacting with VgrGs, such as 72‐kDa TseL (Dong *et al*, 2013), in agreement with the model that many effectors bind to the VgrG/PAAR spike (Fig EV6; Shneider *et al*, 2013). However, the repertoire of secreted effectors is large (Durand *et al*, 2014; Alcoforado Diniz *et al*, 2015; Hachani *et al*, 2016), and therefore, it is likely that the overall shape of T6SS baseplate will vary between organisms. This could be achieved either by changes in the angle between TssF/G and TssK, or elongating the wedge or TssK assembly with a help of flexible C‐terminal head domain (Nguyen *et al*, 2017). Our reconstruction also suggests that TssK protein may be involved in recruitment of certain effectors to the spike. Additionally, it is possible that some organisms evolved proteins that would serve as extensions for the wedge and would thus further increase the size of the baseplate cavity similarly to the variations in baseplate sizes seen in phages (Fu *et al*, 2011; Schwarzer *et al*, 2012; Hu *et al*, 2015; Büttner *et al*, 2016; Nováček *et al*, 2016; Taylor *et al*, 2016).

The cryo‐electron microscopy reconstruction of the whole baseplate, sheath–tube, and distal end presented here provides an overall view of the system and will serve as a scaffold for placing high‐resolution structures of individual T6SS components or their complexes. The approach used here can in principle be used to further improve the final resolution by collecting a significantly larger dataset. However, in contrast to phages, T6SS evolved into a highly dynamic nanomachine undergoing constant assembly and disassembly. Therefore, it is likely that the T6SS baseplate and cap are partially unstable during isolation. Moreover, even when limiting Hcp expression, most of each isolated T6SS complex is composed of the extended sheath–tube polymer, which further limits the number of particles that can be detected per single micrograph, thus complicating data collection. Additionally, particle heterogeneity and dynamic nature of the distal end and possibly of the baseplate decrease the final resolution and chances to resolve all structural features, which prevent us to unambiguously assign proteins to the densities. Nevertheless, a combination of mutagenesis, live‐cell imaging, *in situ* cryo‐electron tomography, and availability of atomic resolution models will eventually provide a complete model of T6SS mode of action.

## Materials and Methods

### VipA/VipB sheath preparation

Sheath preparation was done as described previously (Brackmann *et al*, 2017). Briefly, overnight cultures were diluted and regrown in fresh LB to a final optical density at 600 nm of 1. Cells were collected, resuspended, and lysed. After removal of cell debris, the supernatant was subjected to ultraspeed centrifugation in order to collect VipA/VipB sheath. The sample containing sheath was washed and then used for SDS–PAGE.

Preparation of sheath from Hcp‐limited cells was done with following modifications: After reaching an optical density at 600 nm of 1, arabinose was added to the cells in a final concentration of 0.02% to induce Hcp expression. Cells were centrifuged immediately for 6 min at 5,000 × *g* and room temperature, resuspended, and lysed. Ultraspeed centrifugation and washing steps were performed as described previously (Brackmann *et al*, 2017).

### Fluorescence microscopy

Fluorescence microscopy and image processing were carried out as described previously (Brackmann *et al*, 2017). Briefly, for *hcp* complementation experiments, overnight cultures were washed once in LB, diluted 1:100 into fresh medium supplemented with appropriated antibiotics, and cultivated to an optical density (OD) at 600 nm of 1. Cells were concentrated to OD 10, subsequently spotted on a LB 1% agarose pad containing 0.02% L‐arabinose, and covered with a glass coverslip. Bacteria were directly imaged for 30–40 min at 25°C. To carry out fluorescence microscopy experiments, we used a Nikon Ti‐E‐inverted motorized microscope with Perfect Focus System and Plan Apo 100× Oil Ph3 DM (NA 1.4) objective lens. SPECTRA X light engine (Lumencor) and ET‐GFP (Chroma #49002) filter set was used to excite and filter fluorescence. The setup further contained a sCMOS camera pco.edge 4.2 (PCO, Germany) (pixel size 65 nm) and VisiView software (Visitron Systems, Germany) to record images. Temperature was set to 30°C, and humidity was regulated to 95% by an Okolab T‐unit (Okolab). Fiji (Schindelin *et al*, 2012) was used for additional image processing as described previously (Basler *et al*, 2013).

### Mass spectrometry

Mass spectrometry was carried out as described previously (Brackmann *et al*, 2017). Briefly, samples were dissolved in TN‐buffer, reduced, and alkylated. Proteins were digested overnight and supplemented with TFA to a final concentration of 1%. Peptides were cleaned up using PreOmics Cartridges (PreOmics, Martinsried, Germany) following the manufacturer's instructions. After drying the samples under vacuum, the peptides were resuspended in 0.1% aqueous formic acid solution at a concentration of 0.5 mg/ml. 0.5 μg of peptides of each sample was subjected to LC‐MS analysis as described previously (Brackmann *et al*, 2017).

MS1 and MS2 scans were acquired at a target setting of 1E6 ions and 10,000 ions, respectively. The collision energy was set to 35%, and one microscan was acquired for each spectrum. All raw files acquired by DDA were converted to mgf format using msconvert (version 3.0, ProteoWizard, http://proteowizard.sourceforge.net/). The files were searched against a decoy (consisting of forward and reverse protein sequences) database of predicted protein sequence of *V. cholerae* (Uniprot, Organism ID: 243277, download date 11/07/2016, containing known contaminants, resulting in a total of 3,784 proteins) using Mascot (Matrix Science, version 2.4). The search parameters were set as follows: full tryptic specificity was required (cleavage after lysine and arginine residues unless followed by proline); up to three missed cleavages were allowed; carbamidomethyl (C) was set as a fixed modification; oxidation (M) and acetyl (Protein N‐term) were set as variable modifications; 10 ppm precursor mass tolerance; and 0.6‐Da fragment mass tolerance for CID tandem mass spectra. After importing the result files to Scaffold (http://www.proteomesoftware.com, version 4), the FDR rate was set to < 1% for protein identifications by the local Scaffold FDR algorithm based on the number of decoy hits.

### Negative‐stained sample preparation, data acquisition, and image processing

An aliquot of 3 μl of sheath sample was applied onto freshly glow‐discharged carbon‐coated 300‐mesh copper grid, blotted, washed with 10 μl of TN‐buffer, blotted again, and stained with 2% uranyl acetate for 10 s. 200 images were acquired using CM200FEG microscope (Philips) on TVIPS F416 CMOS camera, operated at 200 kV at a nominal magnification of 38,000×, corresponding to pixel size of 2.81 Å. Contrast transfer function for each micrograph was estimated using CTFFIND4 (Rohou & Grigorieff, 2015). 2,248 particles corresponding to the ends of extended T6SS assemblies were picked using XMIPP manual picking utility within SCIPION framework (de la Rosa‐Trevín *et al*, 2016). Extracted particles were phase‐flipped and subjected for reference‐free 2D classification without CTF correction in RELION1.4 (Scheres, 2012).

### Cryo‐EM sample preparation and data acquisition

An aliquot of 3 μl of sheath sample was applied onto freshly glow‐discharged Quantifoil R2/1 holey carbon grids (Quantifoil Micro Tools GmbH, Germany), blotted for 3 s, and vitrified using a Vitrobot MK4 (FEI Corp., The Netherlands). The chamber was maintained at 4°C and 100% humidity during the blotting process.

Data of the isolated T6SS assemblies were acquired using Titan Krios microscope (FEI Corp.) equipped with an energy filter (slit width 20 eV) on a K2 Summit direct electron detector (Gatan Inc., USA) in counting mode, operated at 300 kV and at a nominal magnification of 130,000×, corresponding to a calibrated pixel size of 1.06 Å, and a defocus ranging from 1.5 to 3 μm. 9,202 movie series were collected automatically using the SerialEM software (Mastronarde, 2005). For each movie, 40‐frame exposures were taken at 0.4 s per frame (16 s total exposure time), using a dose rate of 5e‐/pixel/s.

### Image processing

Movie frames were aligned using MotionCorr2 (Zheng *et al*, 2017) to correct for specimen motion. The averages of the aligned frames were used for data processing within SCIPION 1.0.1 (de la Rosa‐Trevín *et al*, 2016). The contrast transfer function of each micrograph was estimated using the Gctf 1.06 program (Zhang, 2016). Baseplates and distal ends were manually selected from the micrographs using XMIPP manual picking utility in SCIPION 1.0.1 and extracted with a box size of 512 pixels. Particles were binned to have the box size of 256 pixels, corresponding to the pixel size of 2.12 Å. 21,446 baseplate and distal‐end particles were classified into 20 classes using reference‐free 2D classification with RELION1.4. After 2D classification, a total of 2,660 baseplates and 3,710 distal ends were used for 3D refinement and classification. Best baseplate and distal‐end class averages were used for initial volume estimation using XMIPP RANSAC protocol in SCIPION (Vargas *et al*, 2014). Resulted volumes were low‐pass‐filtered to 60 Å and used as a reference model for 3D auto‐refinement. C6 symmetry was imposed during 3D refinement. Better‐resolved rigid sheath densities from baseplate and distal‐end reconstructions were used for the soft mask creation for subsequent focused 3D refinement with small local angular sampling. Finally, refined particles and model were imported into RELION2.1 (Kimanius *et al*, 2016) for the auto‐refinement using solvent‐flattened FSCs. This procedure is suggested for elongated particles, when the protein complex represents a relatively small fraction of the reconstructed volume. This procedure resulted in 8.7 Å resolution baseplate and 7.5 Å resolution distal‐end reconstructions. Tight mask around baseplate wedge and first sheath ring was created and focused 3D classification, and further refinement of the best class with 1,265 baseplate particles was performed with RELION2.1, which resulted in resolution improvement from 8.5 to 8 Å.

Straightforward baseplate reconstruction with C3 symmetry resulted in a map with strong artifacts. These artifacts most likely appeared due to the six‐start sheath helix, occupying majority of the volume inside the particle. Instead, symmetry relaxation from C6 to C3 was performed with *relion_particle_symmetry_expand* utility from RELION2.1, followed by masked 3D classification without alignment. Smooth soft mask lacking sixfold‐related features was created around the baseplate region. The resulting 11 Å resolution model showed the same features as a sixfold symmetrized model and trimeric features of the VgrG spike. Local resolution variations of the baseplate and distal‐end maps were estimated with Resmap (Kucukelbir *et al*, 2014).

To perform focused refinement of the connector protein region, we low‐pass filtered the refined baseplate map to 80 Å and generated a small mask around TssK region with Segger (Pintilie *et al*, 2010) in UCSF Chimera (Pettersen *et al*, 2004; Goddard *et al*, 2007). Mask was prepared for RELION2.1 with *relion_mask_create* with extended width and soft edge of five pixels. The subsequent masked 3D classification without alignment and focused 3D refinement of the best 3D class (Scheres, 2016) resulted in the connector protein density of 10 Å resolution.

### Models segmentation and interpretation

Baseplate and distal‐end reconstructions were rendered, segmented, and interpreted using UCSF Chimera (Pettersen *et al*, 2004; Pintilie *et al*, 2010). Volume‐to‐mass scale coefficient was calculated for the proteins with available X‐ray crystallographic structure (Leiman *et al*, 2010).

### Accession numbers

The EM map was deposited to EMDB (http://www.emdatabank.org) with accession number for the single‐particle reconstruction of a distal‐end EMD‐3878, single‐particle reconstruction of a baseplate EMD‐3879.

## Author contributions

SN collected electron microscopy data, performed image processing, and data analysis. MBr isolated and purified the sheaths and contributed to data collection and analysis. JPS isolated and purified sheaths from Hcp‐limited cells and performed MS data collection and analysis. JPS performed imaging and image processing of Hcp‐limited cells. KNG and HS provided support with and supervised data collection. MBa conceived the project and analyzed the data. SN and MBa wrote the manuscript. All authors read the manuscript.

## Conflict of interest

The authors declare that they have no conflict of interest.

## Supporting information



Appendix

Expanded View Figures PDF

Table EV1

Table EV2

Table EV3

Dataset EV4

Dataset EV5

Dataset EV6

Dataset EV7

Review Process File
