# Supplementary material for: Cryo‐EM reconstruction of Type VI secretion system baseplate and sheath distal end
Source: EMBO J. 2017 Dec 18;37(4):e97103. doi: 10.15252/embj.201797103 (PMC5813253; doi:10.15252/embj.201797103)
Supplement: Supplementary file 1 — Appendix [file EMBJ-37-e97103-s001.pdf]

Table of contents

- Appendix Figure S1. **Symmetry analysis, localized connector refinement and homology to T4 gp10.**
- Appendix Figure S2. **Comparison of the T6SS baseplate with the T4 phage inner baseplate.**
- Appendix Figure S3. **Schematic representation of the genomic region encoding T6SS from *Vibrio cholerae*.**
- Appendix Table S1. **Cryo-EM data collection and processing.**
- Appendix Table S2. **Fitting of X-ray crystallographic structures into cryo-EM density maps of baseplate and cap.**
- Appendix Table S3. **Strains used in this study, related to Material and Methods.**

Appendix Fig. S1

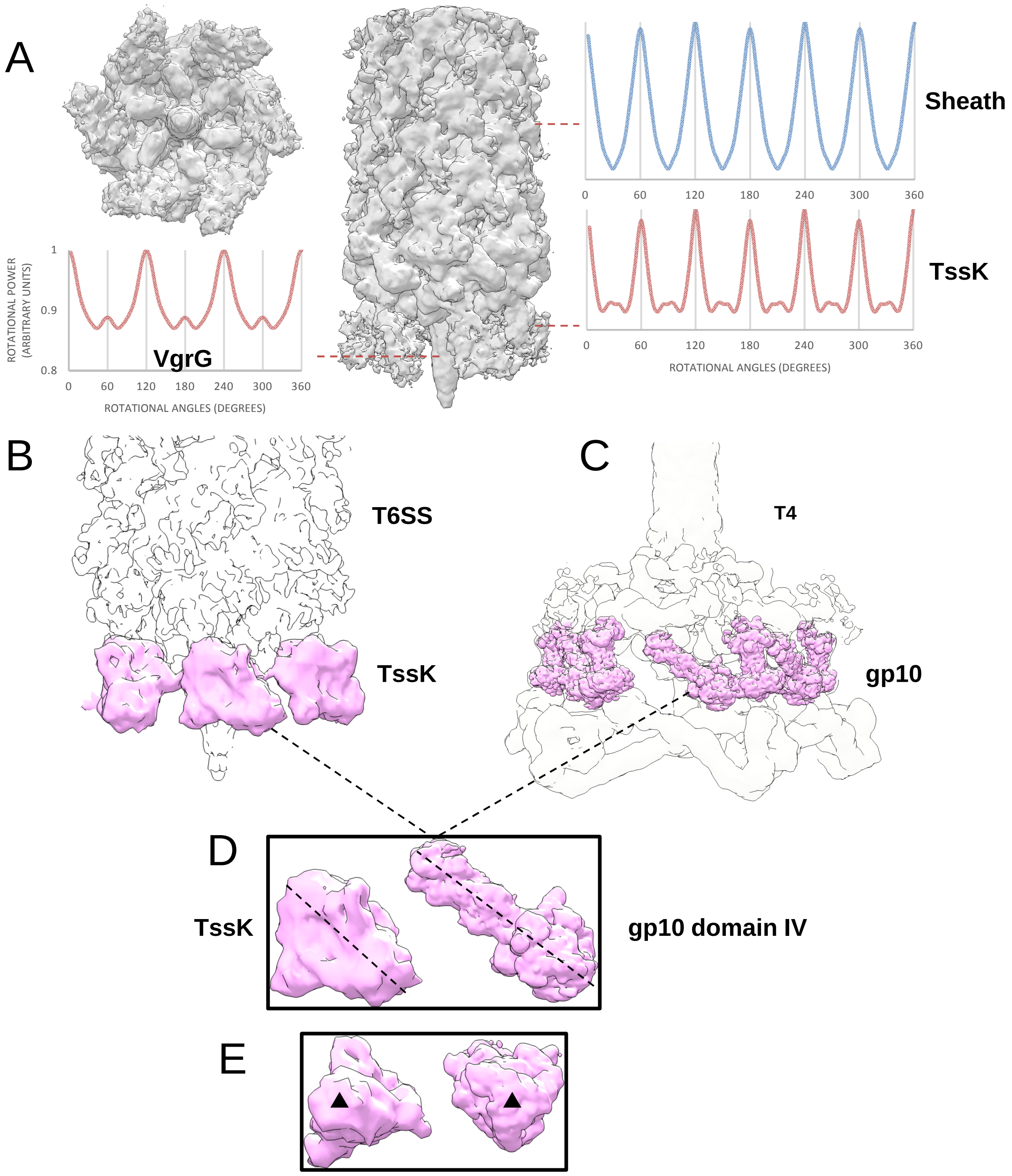

Symmetry analysis, localized connector refinement and homology to T4 gp10.

(A). Bottom and side views of the 3-fold averaged sheath-baseplate reconstruction. Rotational power spectra of the cross sections through the sheath, wedge, and spike regions.

(B). Tilted view of the composite baseplate cryo-EM reconstruction, except for the disordered connector density, replaced by six copies of locally refined reconstruction colored in pink.

(C). Tilted view of the 12 Å T4 baseplate cryo-EM reconstruction (EMD-1048), with fitted upper peripheral baseplate (EMD-3394) colored in pink.

(D). Side views of the TssK connector protein and domain IV of T4 gp10 with three-fold symmetry axis highlighted with black dashed lines.

(E). View along three-fold symmetry axis of (D).

Appendix Fig. S2

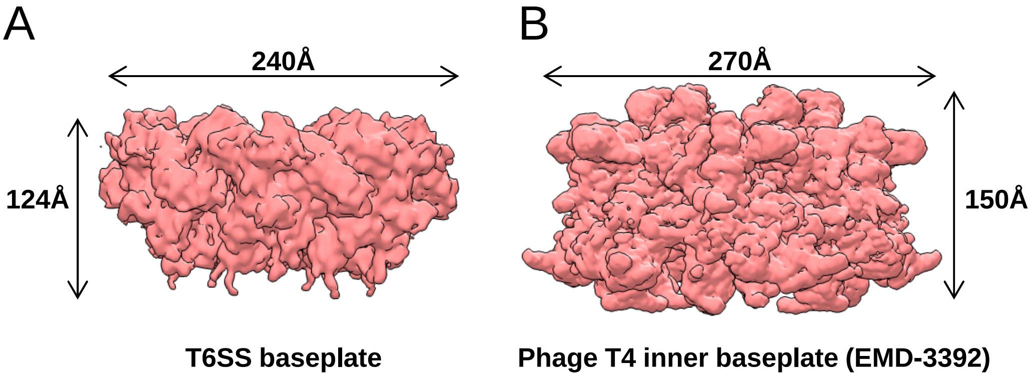

Comparison of the T6SS baseplate with the T4 phage inner baseplate.

(A). Extracted putative baseplate of 8 Å T6SS sheath-baseplate reconstruction.

(B). Cryo-EM reconstruction of the T4 inner baseplate (EMD-3392) low-pass filtered to 8 Å**.**

Appendix Fig. S3

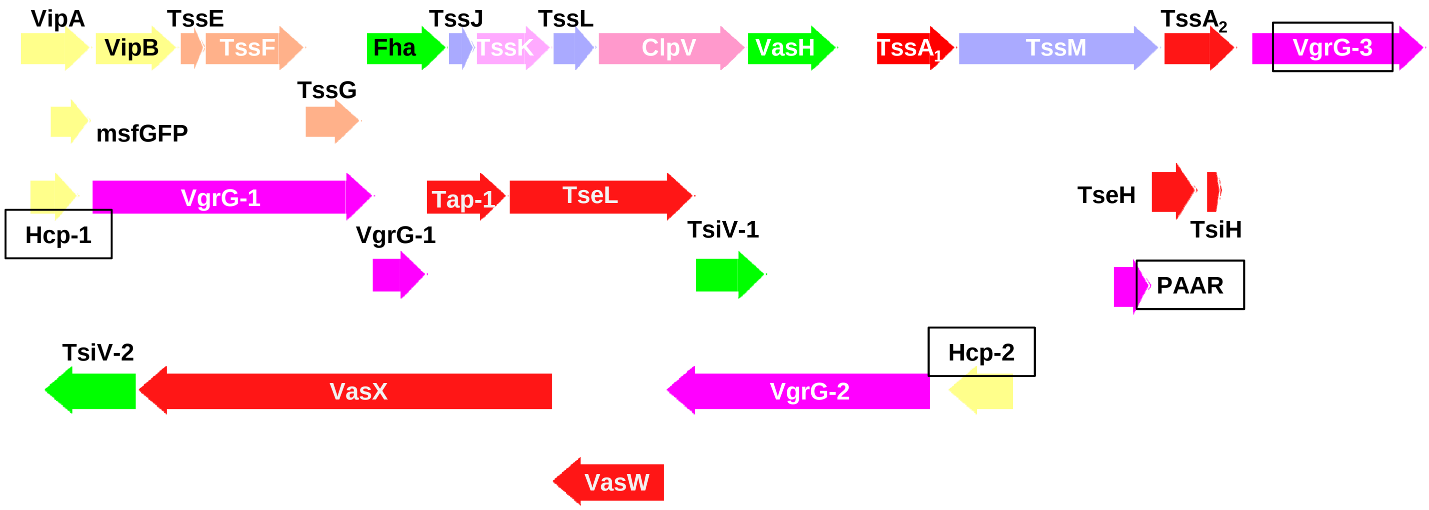

Schematic representation of the genomic region encoding T6SS from *Vibrio cholerae*.

Main cluster with VgrG-3, and three auxiliary clusters – Hcp-1, Hcp-2 and PAAR are shown. Baseplate, membrane complex, cap, effectors, adaptors, immunity and regulatory proteins are shown.

Appendix Table S1. Cryo-EM data collection and processing.

T6S Part	Symmetry	Movies		Particles						Pixel size, Å	Resolution, Å	Software
		Non Hcp lim.	Hcp lim.	Non Hcp lim.			Hcp lim.		Final 3DR Combined			
				Picked	After 2D	3D	Picked	After 2D				
**Baseplate**	C6/C3	6,603	2,599	21,446	8,309	2,660	12,832	3,241	1,265	2.12	8/11	MotionCorr2, Gctf, Scipion, RELION1.4, 2
**Distal end**	C6					3,710			3,710*		7.5	
**TssK**	C6-C1 relaxation	-							3,600		10	RELION2

* Final 3D refinement with particles from non-Hcp limited cells only.

Appendix Table S2. Fitting of X-ray crystallographic structures into cryo-EM density maps of baseplate and distal end.

Model	PDB	% atoms	Correlation coefficient
Sheath ring 1	5MXN	87	0.87
Ring 1 refitted		90	0.89
Ring 2		86	0.86
Ring 3		89	0.88
Ring 4		91	0.89
Ring 5		91	0.90
VgrG/PAAR C6	4MTK/4JIV	88	0.88
VgrG/PAAR C3		94	0.93
Ring N (topmost)	5MXN	76	0.79
Ring N refitted		84	0.86
Ring (N-1)		90	0.87
Hcp N (topmost)	5MXN*	60	0.87
Hcp N-1		77	0.90
Hcp N-2		90	0.95
VipA/VipB only into distal end	5MXN**	73	0.82
TssK	5M30	66	0.92

* Hcp tube and **VipA/VipB sheath models were extracted from complete sheath-tube model (PDB 5MXN).

Appendix Table S3. Strains used in this study, related to Material and Methods.

Organism	Genotype	Plasmid	Relevant features	Source
V. cholerae 2740-80	lacZ^{-}, Str^{r}, *vipA-msfGFP*		C-terminal chromosomal fusion of *msfGFP* to *vipA*	(Kudryashev et al., 2015)
	lacZ^{-}, Str^{r}, *ΔvipA, ΔflgG*	pBAD24-*vipA*	Complementation of *vipA* deletion from inducible vector; Amp^{r}	(Brackmann et al., 2017)
	lacZ^{-}, Str^{r}, *ΔvipA, ΔflgG*	pBAD24-*vipA-N3*	Complementation of *vipA* deletion from inducible vector; Amp^{r}	
	lacZ^{-}, Str^{r}, *vipA-msfGFP, Δhcp1, Δhcp2*		Deletion of both *hcp* variants in *vipA-msfGFP* background	(Vettiger et al., 2016)
	lacZ^{-}, Str^{r}, *vipA-msfGFP, Δhcp1, Δhcp2, ΔflgG*		*flgG* deletion in *hcp* mutant background	this study
	lacZ^{-}, Str^{r}, *vipA-msfGFP, Δhcp1, Δhcp2, ΔflgG*	pBAD24-*hcp2*	Complementation of *hcp* deletion from inducible vector; Amp^{r}	
	lacZ^{-}, Str^{r}, *vipA-N3-msfGFP, Δhcp1, Δhcp2, ΔflgG*		Chromosomal integration of *vipA-N3-msfGFP* in *hcp / flgG* deletion background	
	lacZ^{-}, Str^{r}, *vipA-N3-msfGFP, Δhcp1, Δhcp2, ΔflgG*	pBAD24-*hcp2*	Complementation of *hcp* deletion in *vipA-N3-msfGFP* background from inducible vector; Amp^{r}	
E. coli SM10 λ pir	Km^{r}, thi-1, thr, leu, tonA, lacY, supE, recA::RP4-2-Tc::Mu, pir	pWM91	Allelic replacement vector used for all in-frame deletions by conjugation; *sacB*, Amp^{r}/Gent^{r}	
DH5α λ pir	F^{-}, endA1, glnV44, thi-1, recA1, relA1, gyrA96 deoR, nupG, Φ80d*lacZ*ΔM15, (*lacZYA-argF*)U169, hsdR17(rK^{-} mK^{+}), λ^{-}		Cloning strain	
