# Supplementary material for: Cryo‐EM reconstruction of Type VI secretion system baseplate and sheath distal end
Source: EMBO J. 2017 Dec 18;37(4):e97103. doi: 10.15252/embj.201797103 (PMC5813253; doi:10.15252/embj.201797103)
Supplement: Supplementary file 2 — Expanded View Figures PDF [file EMBJ-37-e97103-s002.pdf]

Expanded View Figures

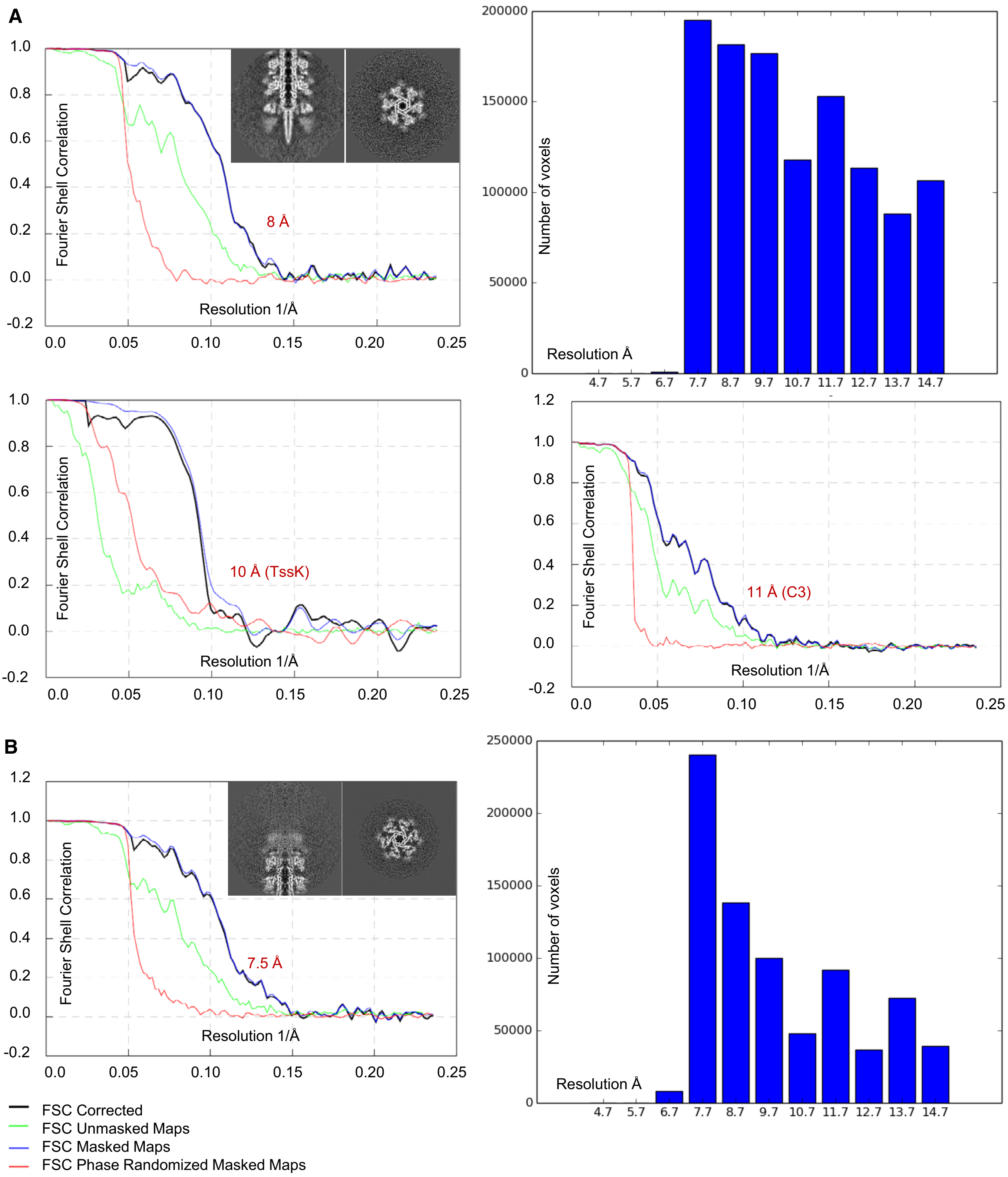

Figure EV1.

◀ **Figure EV1. Analysis of raw data, estimation of resolution.**

A Representative slices through the raw sheath–baseplate reconstruction. Gold-standard FSC curves calculated for the sixfold-averaged (top left) and threefold-averaged (bottom right) baseplate reconstructions and for the TssK reconstruction (bottom left). Histogram of the local resolution estimation of the baseplate reconstruction (top right).

B Representative slices through the raw sheath–distal-end reconstruction. Left: Gold-standard FSC curves calculated for the sixfold-averaged distal-end reconstruction. Right: Histogram of the local resolution estimation of the distal-end reconstruction.

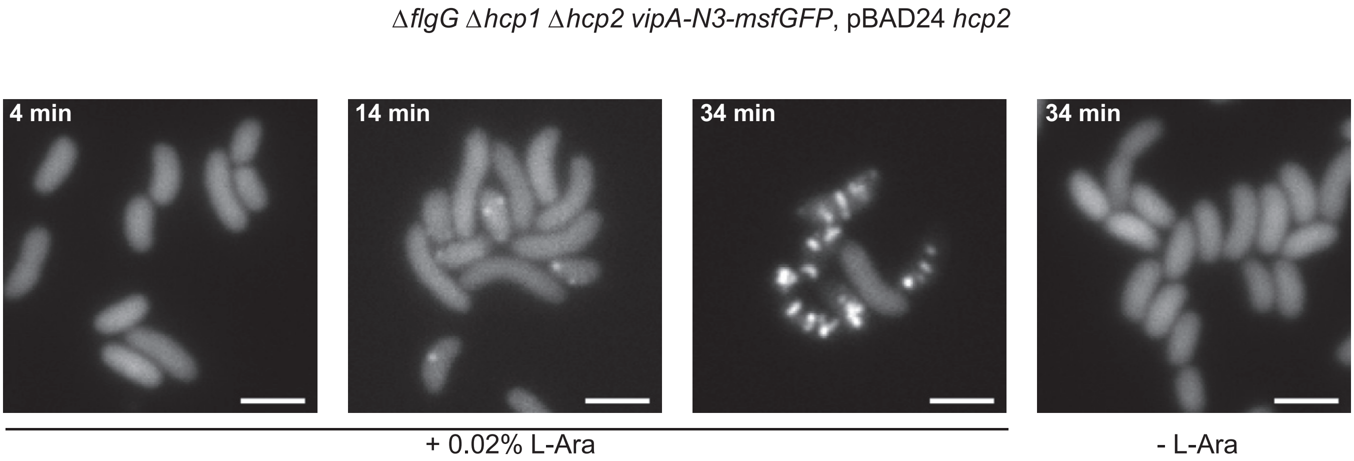

Figure EV2. Sheath formation in Hcp-limited cells.

Fluorescence timelapse images of *Vibrio cholerae vipA-N3-msfGFP* in *hcp1/2* mutant background, complemented with *hcp* expressed from L-arabinose-inducible vector pBAD24. Scale bars are 2 μm.

 © 2017 The Authors

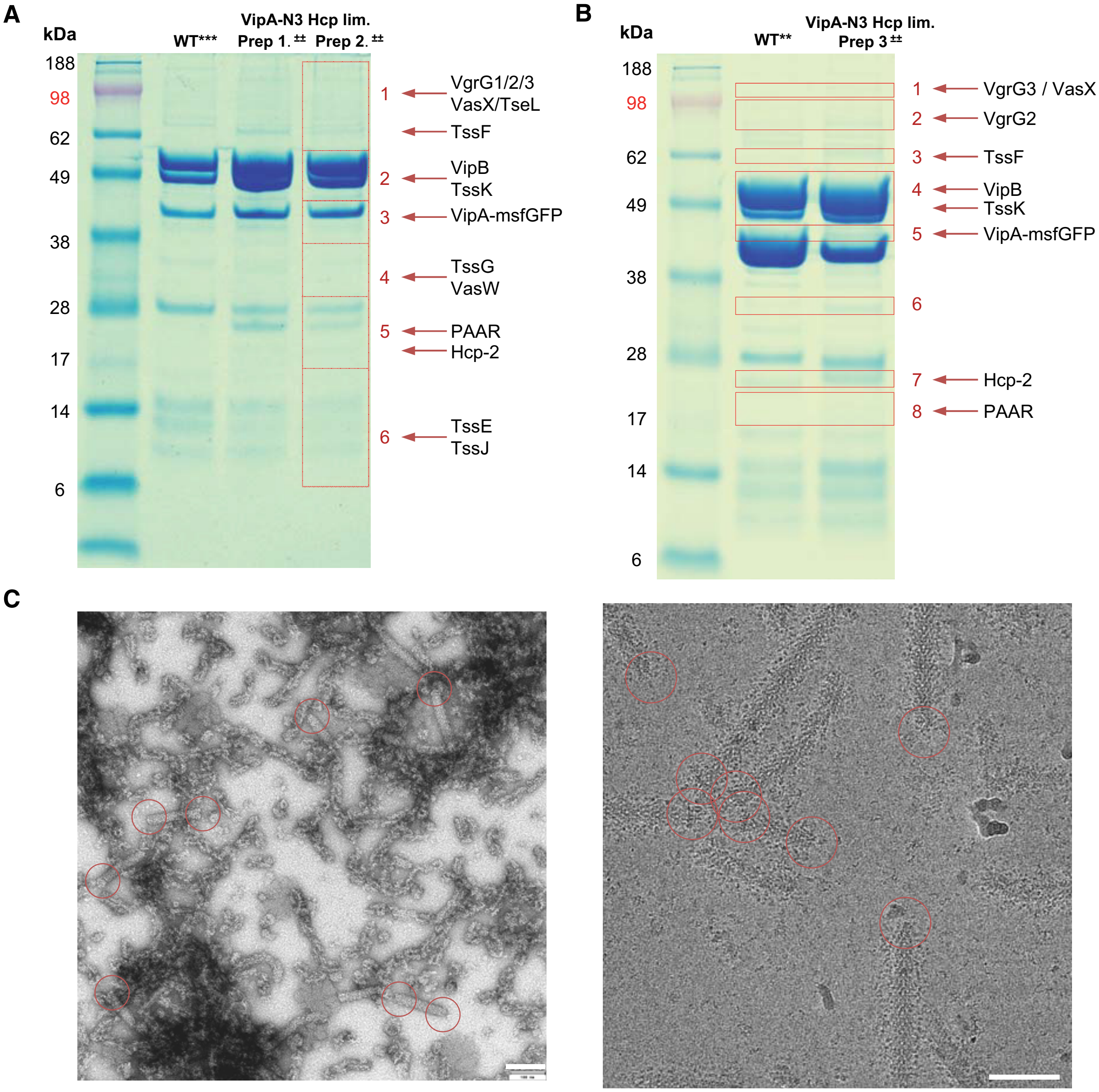

Figure EV3. Mass spectrometry and EM analysis of preparations from Hcp-limited cells.

A SDS–PAGE of purified sheaths: wild-type Hcp-limited prep, and (Hcp-limited + VipA-N3) Prep1 and Prep2. Areas 1–6 cut from gel and sent for mass spectrometry. Detected structural proteins are shown on the right.

B SDS–PAGE of purified sheaths: wild-type and (Hcp-limited + VipA-N3) Prep3. Areas 1–8 cut from gel and sent for mass spectrometry. Detected structural proteins are shown on the right.

C Raw negative-stained and cryo-EM images of sample from (A) (Prep2). Scale bars are 100 nm. Representative baseplates and distal ends in the field of view are outlined with red circles.

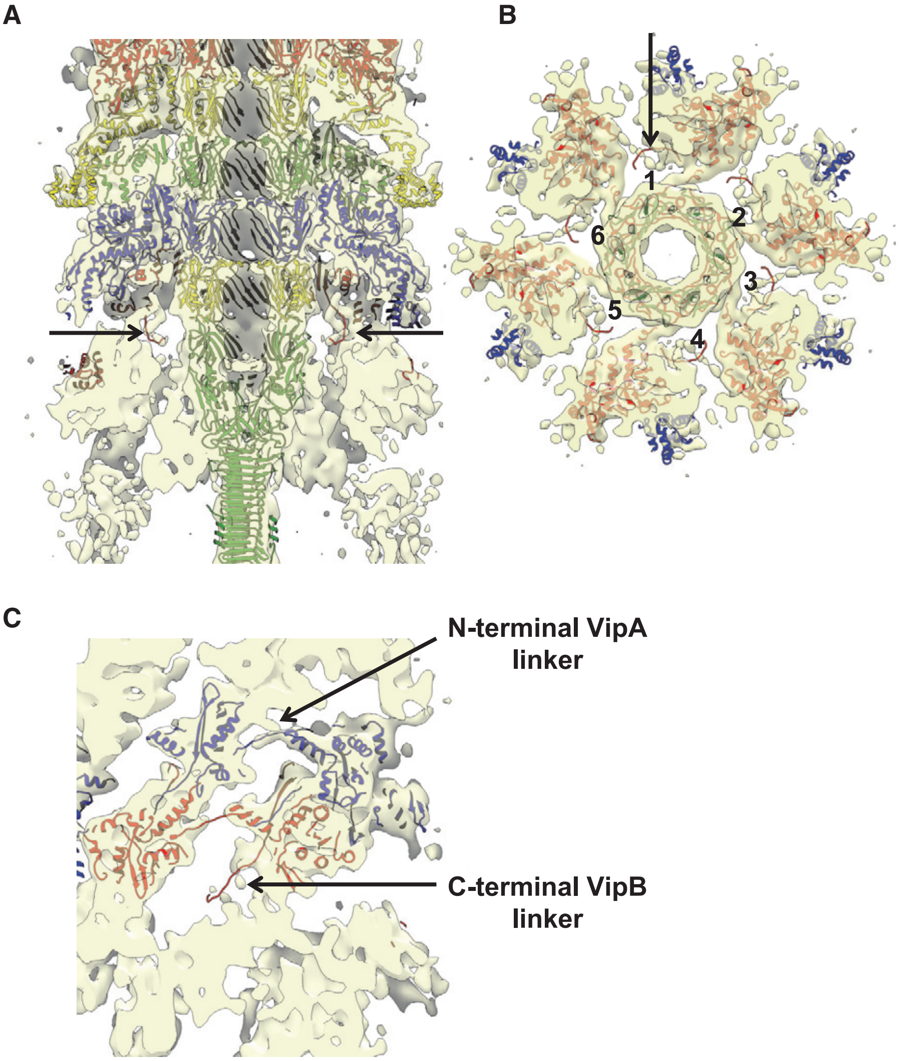

Figure EV4. Baseplate–sheath connection.

A Side cutaway view of the baseplate reconstruction with fitted atomic model of VipA-N3 sheath–tube (PDB 5MXN) and VgrG-1 trimer (PDB 4MTK) and PAAR monomer (PDB 4JIV). Putative C-terminal VipB linkers are highlighted with black arrows.

B Bottom cutaway view of (A) below the first sheath ring. Six putative C-terminal VipB linkers are numbered, and one is highlighted with black arrow.

C Enlarged side cutaway view of (A) of the first and second sheath rings with fitted atomic model of VipA-N3 sheath–tube (PDB 5MXN). Putative C-terminal VipB and N-terminal VipA linkers are highlighted with arrows. N-terminal linker connects to the neighboring protomer in the same ring.

 © 2017 The Authors

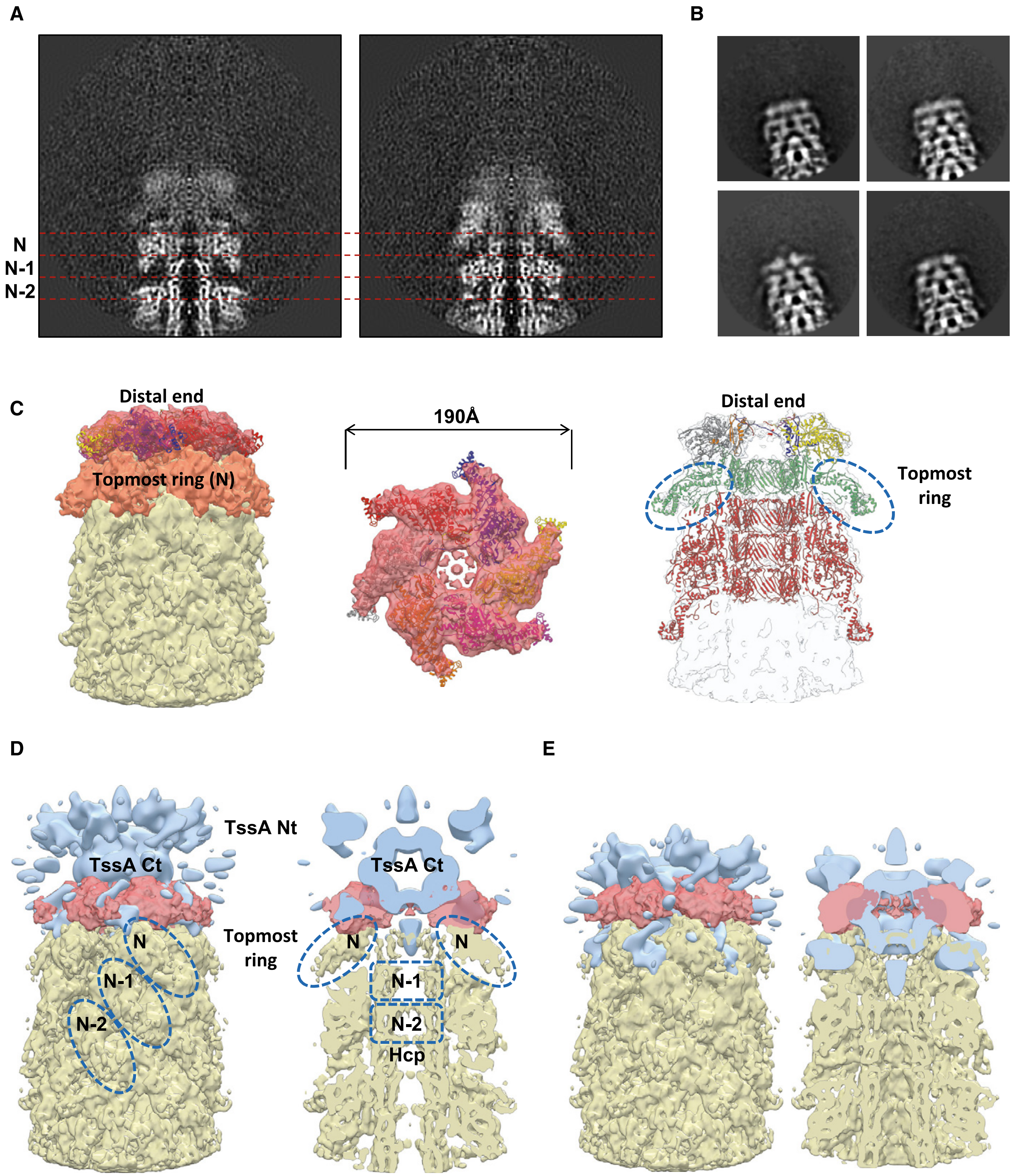
A
N
N-1
N-2
B
C
Distal end
Topmost ring (N)
190Å
Distal end
Topmost
ring
D
TssA Nt
TssA Ct
Topmost
ring
N
N-1
N-2
TssA Ct
N
N
N-1
N-2
Hcp
E

Figure EV5.

◀

Figure EV5. Morphology of the sheath distal end.

A Two orthogonal slices through the raw sheath–distal-end reconstruction shown. Ring numbers N, (N−1), and (N−2) correspond to the topmost ring and the two previous rings, and are separated by dashed red lines.

B Representative reference-free 2D class averages of the distal-end particles, extracted from cryo-EM images.

C Left and center: Side and top views of putative docking of refitted sheath domain of the VipA-N3 sheath–tube (PDB 5MXN) into distal end. Right: Putative pseudo atomic model of the distal end with refitted sheath domain of the VipA-N3 sheath–tube, refitted topmost sheath–tube ring and three rings below (PDB 5MXN). Topmost sheath rings are outlined with dashed blue circles.

D, E Putative dockings of low-resolution EM reconstruction EAEC TssA (EMD-3282) into distal end of T6SS. Topmost sheath ring N, two previous sheath rings (N-1) and (N-2), and two tube rings (N-1) and (N-2) are outlined with dashed blue circles.

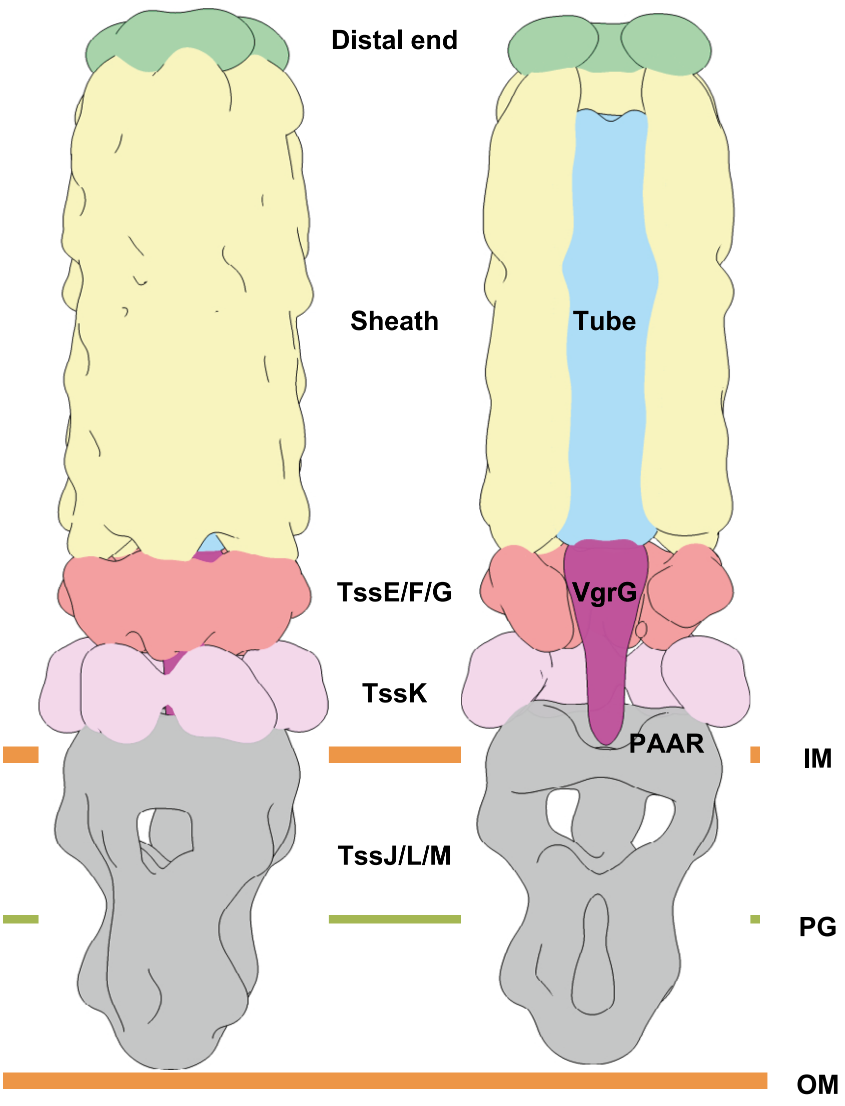

Figure EV6. Schematic representation of the assembled T6SS.
Side and cutaway views of the map, composed of the overlapping sixfold-averaged sheath baseplate, sixfold-averaged sheath–distal end, and fivefold-averaged membrane complex (EMD-2927) reconstructions, strongly filtered with Gaussian filter to 10σ. The map is colored according to putative protein complexes.

 © 2017 The Authors
