# Supplementary material for: Cryo‐EM reconstruction of Type VI secretion system baseplate and sheath distal end
Source: EMBO J. 2017 Dec 18;37(4):e97103. doi: 10.15252/embj.201797103 (PMC5813253; doi:10.15252/embj.201797103)
Supplement: Supplementary file 3 — Table EV1 [file EMBJ-37-e97103-s003.docx]

**Table EV1.** Summary of MS analysis. (Semi-)quantitative comparison of sheath preparations from WT*, VipA-N3± (Brackmann et al., 2017), and VipA-N3 Hcp-limited cells±±. Areas 1-6 cut from gel (Fig. EV3 a). QV—Quantitative value (normalized total spectra), PC—percent coverage. Percent coverage threshold was set to 10%. Full list of identified proteins is in Dataset EV4.

| **T6 part** | **WT*** | | **VipA-N3^±^** | | **VipA-N3 Hcp lim. Prep 2 ^±±^** | |
| --- | --- | --- | --- | --- | --- | --- |
|  | QV | PC | QV | PC | QV | PC |
| **Sheath/Tube** | | | | | | |
| VipB | 61 | 59% | 71 | 66% | 1002 | 88% |
| VipA | 68 | 91% | 37 | 74% | 413 | 89% |
| Hcp-2 | 4 | 43% | 24 | 54% | 81 | 61% |
| **Baseplate** | | | | | | |
| TssE |  |  | 1 | 25% | 20 | 46% |
| TssF | 3 | 13% | 8 | 35% | 63 | 36% |
| TssG |  |  | 4 | 27% | 69 | 44% |
| TssK | 7 | 30% | 9 | 49% | 168 | 67% |
| VgrG1 |  |  | 9 | 19% | 47 | 34% |
| VgrG2 | 3 | 13% | 11 | 39% | 62 | 61% |
| VgrG3 | 5 | 16% | 15 | 40% | 60 | 47% |
| PAAR |  |  |  |  | 14 | 29% |
| **Effector/Adaptor** | | | | | | |
| VasX | 5 | 11% | 7 | 18% | 41 | 37% |
| VasW |  |  |  |  | 9 | 17% |
| TseL |  |  |  |  | 7 | 15% |
| **Membrane complex** | | | | | | |
| TssJ |  |  |  |  | 5 | 17% |
| **Other** |  |  |  |  |  |  |
| Fha | 8 | 38% | 6 | 31% | 4 | 21% |

QV = (Average of the spectrum counts for all of the samples) * (Spectrum counts in each sample) / (Individual sample’s sum)

PC = The percentage of all the amino acids in the protein sequence that were covered by identified peptides detected in the sample

* lacZ‐, Strr, ∆vipA, ∆flgG, pBAD24-vipA (WT)

± lacZ‐, Strr, ∆vipA, ∆flgG, pBAD24-vipA-N3

±± lacZ‐, Strr, vipA-N3-msfGFP, ∆hcp1, ∆hcp2, ∆flgG, pBAD24-hcp2
