# Supplementary material for: Cryo‐EM reconstruction of Type VI secretion system baseplate and sheath distal end
Source: EMBO J. 2017 Dec 18;37(4):e97103. doi: 10.15252/embj.201797103 (PMC5813253; doi:10.15252/embj.201797103)
Supplement: Supplementary file 4 — Table EV2 [file EMBJ-37-e97103-s004.docx]

**Table EV2.** Summary of MS analysis. (Semi-)quantitative comparison of sheath preparations from WT** and VipA-N3 Hcp±±-limited cells. Areas 1-8 cut from gel (Fig. EV3 b). QV—Quantitative value (normalized total spectra), PC—percent coverage. Percent coverage threshold was set to 10%. Full list of identified proteins is in Dataset EV5.

| **T6 part** | **WT**** | | **VipA-N3 Hcp lim. Prep 3 ^±±^** | |
| --- | --- | --- | --- | --- |
|  | QV | PC | QV | PC |
| **Sheath / Tube** | | | | |
| VipB | 98 | 85% | 85 | 78% |
| VipA | 54 | 70% | 58 | 86% |
| Hcp-2 |  |  | 18 | 21% |
| **Baseplate** | | | | |
| TssF |  |  | 76 | 13% |
| TssK | 3 | 33% | 17 | 54% |
| VgrG2 |  |  | 25 | 18% |
| VgrG3 |  |  | 28 | 15% |
| PAAR |  |  | 15 | 22% |
| **Effector / Adaptor** | | | | |
| VasX |  |  | 39 | 13% |

QV = (Average of the spectrum counts for all of the samples) * (Spectrum counts in each sample) / (Individual sample’s sum)

PC = The percentage of all the amino acids in the protein sequence that were covered by identified peptides detected in the sample

** lacZ‐, Strr, vipA-msfGFP (WT)

±± lacZ‐, Strr, vipA-N3-msfGFP, ∆hcp1, ∆hcp2, ∆flgG, pBAD24-hcp2
