# Supplementary material for: Cryo‐EM reconstruction of Type VI secretion system baseplate and sheath distal end
Source: EMBO J. 2017 Dec 18;37(4):e97103. doi: 10.15252/embj.201797103 (PMC5813253; doi:10.15252/embj.201797103)
Supplement: Supplementary file 5 — Table EV3 [file EMBJ-37-e97103-s005.docx]

**Table EV3.** (Semi-)quantitative LC-MS analysis comparing sheath preparations from Hcp-limited cells*** to VipA-N3 Hcp-limited cells±± and WT** to VipA-N3 Hcp-limited cells±±. QV—Quantitative value (normalized total spectra), PC—percent coverage. Percent coverage threshold was set to 10%. Full lists of identified proteins are in Datasets EV6 and EV7.

| **T6 part** | **WT***** | | **VipA-N3** | | **WT**** | | **VipA-N3 Hcp lim. Prep 3 ^±±^** | |
| --- | --- | --- | --- | --- | --- | --- | --- | --- |
|  |  |  | **Prep 2 ^±±^** | |  |  |  |  |
|  | **Hcp lim.** | | | |  |  |  |  |
|  | QV | PC | QV | PC | QV | PC | QV | PC |
| **Sheath / Tube** | | | | | | | | |
| VipB | 401 | 81% | 370 | 63% | 807 | 83% | 714 | 87% |
| VipA | 214 | 82% | 322 | 71% | 363 | 96% | 263 | 94% |
| Hcp-2 | 5 | 43% | 15 | 44% | 18 | 44% | 66 | 47% |
| **Baseplate** | | | | | | | | |
| TssE | 2 | 23% | 4 | 21% |  |  | 6 | 53% |
| TssF |  |  |  |  |  |  | 28 | 33% |
| TssG |  |  |  |  |  |  | 16 | 47% |
| TssK | 12 | 24% | 20 | 19% | 7 | 12% | 78 | 70% |
| VgrG1 | 18 | 19% |  |  |  |  | 38 | 33% |
| VgrG2 | 25 | 41% | 8 | 12% |  |  | 43 | 74% |
| VgrG3 | 46 | 48% | 15 | 15% |  |  | 49 | 55% |
| PAAR |  |  |  |  |  |  | 2 | 19% |
| **Effector / Adaptor** | | | | | | | | |
| VasX | 8 | 11% |  |  |  |  | 21 | 25% |
| VasW |  |  |  |  |  |  | 6 | 31% |
| **Immunity** | | | | | | | | |
| TsiV1 | 2 | 11% |  |  |  |  |  |  |
| TsiV3 | 2 | 20% |  |  |  |  | 2 | 20% |
| **Membrane complex** | | | | | | | | |
| TssJ | 13 | 22% |  |  | 3 | 13% | 3 | 22% |
| **Other** | | | | | | | | |
| Fha | 7 | 22% |  |  |  |  |  |  |

QV = (Average of the spectrum counts for all of the samples) * (Spectrum counts in each sample) / (Individual sample’s sum)

PC = The percentage of all the amino acids in the protein sequence that were covered by identified peptides detected in the sample

** lacZ‐, Strr, vipA-msfGFP (WT)

±± lacZ‐, Strr, vipA-N3-msfGFP, ∆hcp1, ∆hcp2, ∆flgG, pBAD24-hcp2

*** lacZ‐, Strr, vipA-msfGFP, ∆hcp1, ∆hcp2, ∆flgG, pBAD24-hcp2
